# Protease secretions by the invading blastocyst induce calcium oscillations in endometrial epithelial cells via the protease-activated receptor 2

**DOI:** 10.1186/s12958-023-01085-7

**Published:** 2023-04-15

**Authors:** Aurélie Hennes, Johanna Devroe, Katrien De Clercq, Martina Ciprietti, Katharina Held, Katrien Luyten, Nele Van Ranst, Nina Maenhoudt, Karen Peeraer, Hugo Vankelecom, Thomas Voets, Joris Vriens

**Affiliations:** 1 Laboratory of Endometrium, Endometriosis and Reproductive Medicine, Department of Development and Regeneration, KU Leuven, Herestraat 49 Box 611, 3000 Leuven, Belgium; 2 Laboratory of Ion Channel Research, Department of Cellular and Molecular Medicine, VIB Center for Brain & Disease Research, KU Leuven, Herestraat 49 Box 802, 3000 Leuven, Belgium; 3 Leuven University Fertility Center, University Hospitals Leuven, Herestraat 49, 3000 Leuven, Belgium; 4 Laboratory of Tissue Plasticity in Health and Disease, Cluster of Stem Cell and Developmental Biology, Department of Development and Regeneration, KU Leuven, Herestraat 49 Box 804, 3000 Leuven, Belgium

**Keywords:** Early embryo implantation, Endometrium, Embryo-uterine crosstalk, Calcium microfluorimetry, Protease-activated receptor 2, Serin protease, Trypsin

## Abstract

**Background:**

Early embryo implantation is a complex phenomenon characterized by the presence of an implantation-competent blastocyst and a receptive endometrium. Embryo development and endometrial receptivity must be synchronized and an adequate two-way dialogue between them is necessary for maternal recognition and implantation. Proteases have been described as blastocyst-secreted proteins involved in the hatching process and early implantation events. These enzymes stimulate intracellular calcium signaling pathways in endometrial epithelial cells (EEC). However, the exact molecular players underlying protease-induced calcium signaling, the subsequent downstream signaling pathways and the biological impact of its activation remain elusive.

**Methods:**

To identify gene expression of the receptors and ion channels of interest in human and mouse endometrial epithelial cells, RNA sequencing, RT-qPCR and in situ hybridization experiments were conducted. Calcium microfluorimetric experiments were performed to study their functional expression.

**Results:**

We showed that trypsin evoked intracellular calcium oscillations in EEC of mouse and human, and identified the protease-activated receptor 2 (PAR2) as the molecular entity initiating protease-induced calcium responses in EEC. In addition, this study unraveled the molecular players involved in the downstream signaling of PAR2 by showing that depletion and re-filling of intracellular calcium stores occurs via PLC, IP_3_R and the STIM1/Orai1 complex. Finally, in vitro experiments in the presence of a specific PAR2 agonist evoked an upregulation of the ‘Window of implantation’ markers in human endometrial epithelial cells.

**Conclusions:**

These findings provide new insights into the blastocyst-derived protease signaling and allocate a key role for PAR2 as maternal sensor for signals released by the developing blastocyst.

**Graphical Abstract:**

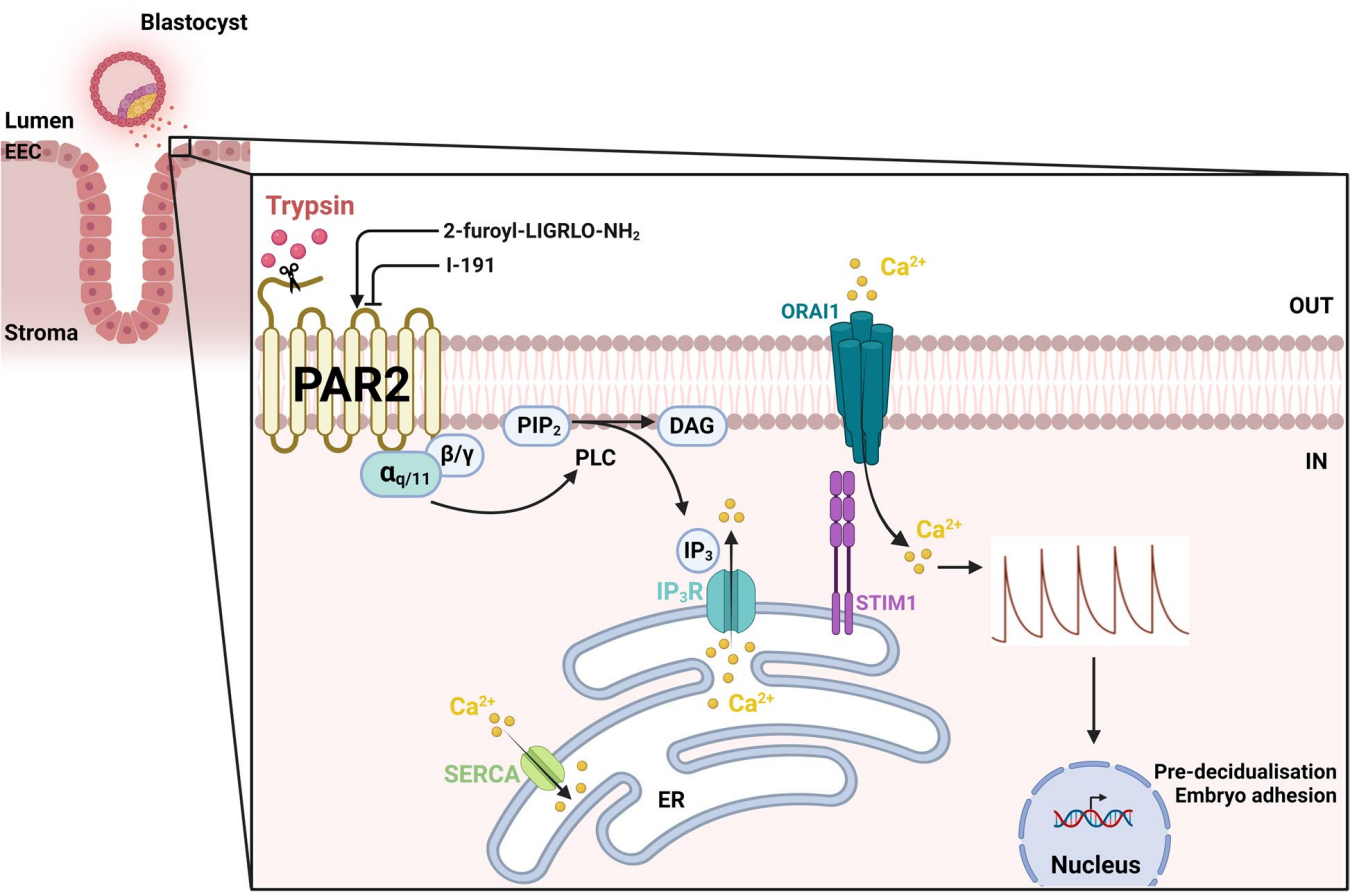

**Supplementary Information:**

The online version contains supplementary material available at 10.1186/s12958-023-01085-7.

## Background

Human fertility is generally considered as a straightforward event, but even in optimal conditions human pregnancy is only achieved in approximately 30% of any given menstrual cycle [1]. One of the reasons that may underly failure of clinical pregnancy is a malfunction at the time of implantation. As embryo implantation requires the conjunction of a competent blastocyst with the receptive endometrium, the coordination of the implantation process via embryo-maternal communication is of utmost importance. During every menstrual cycle, the endometrium undergoes morphological changes that lead to a decidualized state which prepares the endometrium for the invading blastocyst. During a limited timeframe, referred to as the window-of-implantation, the endometrium is able to perceive and translate signals from the contacting and implanting blastocyst to allow invasion of the outgrowing trophoblasts. Indeed, endometrial epithelial cells (EEC) that line the lumen of the uterus are the first to detect signals secreted by the developing blastocyst. The EEC will detect and transduce embryonic signals into downstream signaling pathways, and will render the endometrium more susceptible for the invading blastocyst. In addition, EEC will signal to the underlying endometrial stromal cells (ESC) to maintain and perpetuate the decidualization process, in which stromal cells differentiate into round, secretory, pseudo-epithelial cells that provide nutrients for the invading blastocyst and alter local immunity to allow for proper implantation [2, 3]. Besides Osteopontin (phosphoprotein 1 (SPP1)) and leukaemia inhibitory factor (LIF) [4], expressed by the luminal epithelium to promote adhesiveness, other epithelial markers of the ‘Window of implantation’ (WOI), provided by single-cell transcriptomic research of the human endometrium, include progestagen-associated endometrial protein (PAEP), glutathione peroxidase homolog 3 (GPX3), and C-X-C motif chemokine 14 (CXCL14) [5, 6].

It was shown that pre-implantation blastocysts are capable of secreting several chemical molecules, such as growth factors, hormones and trypsin-like proteases to participate in the hatching process [7]. Recently, attention was given to the involvement of embryo-derived serine proteases in the implantation process [8–10], as it was proposed that developmentally competent blastocysts use serine proteases to signal their competence to the maternal side [11].

Several molecular sensors for the detection of embryonic signals have been described in the EEC. Originally, the epithelial sodium channel (ENaC) was proposed as a potential detector of embryonic signals, initiating intracellular calcium ([Ca^2+^]_I_) changes in response to blastocyst derived trypsin-like proteases [12]. Calcium mobilization in EECs has been described to promote phosphorylation and activation of the transcription factor cAMP response element binding protein (CREB), which results in the upregulation of cyclooxygenase-2 (COX-2) and production of prostaglandin E2 (PGE_2_). PGE_2_ will ultimately activate cAMP-regulated pathways in ESCs and influence the endometrial decidualization process [12]. As a potential mechanism for the increase in [Ca^2+^]_I_, it was suggested that the blastocyst-derived trypsin-like proteases would induce Na^+^ influx and subsequent activation of voltage-dependent Ca^2+^ channels (VDCC) [12]. In later studies, it was declared that Na^+^ entry via ENaC would increase the intracellular Na^+^ concentration and reverse the sodium calcium exchanger (NCX) thereby providing means for Ca^2+^ entry [13]. In addition, the protease-activated receptors (PAR1 and PAR2) were proposed as the main mechanism mediating trypsin-induced [Ca^2+^]_I_ oscillations in Ishikawa cells [13]. Currently, no knowledge exists on the potential interplay between the different Ca^2+^ influx mechanisms to regulate trypsin-induced [Ca^2+^]_I_ oscillations in primary human EEC and the physiological consequences of these [Ca^2+^]_I_ changes on the implantation process are currently unclear.

In this study, we were unable to find evidence for the involvement of the ENaC-VDCC connection nor for the ENaC-NCX link to induce [Ca^2+^]_I_ influxes in EEC. However, we identified PAR2 as a key sensor in the detection of blastocyst signals and its capability to induce [Ca^2+^]_I_ oscillations in primary EEC of both mouse and human. Ultimately, we unraveled the molecular players involved in the downstream signaling of PAR2 and present the first evidence for a biological consequence of PAR2-induced [Ca^2+^]_I_ oscillations in the process of pre-decidualization in human EEC.

## Methods

### Animals

#### Ethical approval

All animal experiments were reviewed and approved by the Ethical Committee for Animal Experiments of KU Leuven, Belgium (P102/2017).

#### Housing

All mice (C57Bl/6 J, 8–12 weeks old) were housed with a maximum of 5 animals per cage under controlled standard conditions with ad libitum access to food pellets and tap water.

#### Progesterone-induced uterine gland knockout (PUGKO) mice model

Female and male C57Bl/6 J mice of 8–12 weeks old were mated. Litters derived from these couples were randomly assigned to the control group or treatment group. Pups assigned to the treatment group received daily progesterone injections (P_4_, 50 µg/g body weight in corn oil) from postnatal day 2 until postnatal day 10 to inhibit uterine gland formation. Pups assigned to the control group were injected with similar volumes of vehicle (corn oil) as the treatment group. Animals were used for experiments at the age of 8–12 weeks.

#### Conditional progesterone specific GCamp3 (F/F) mice

Mice genetically encoded with the Ca^2+^ sensor GCamp3 after a floxed stop codon in the Rosa26 locus were crossed with the progesterone receptor (PR)-cre mice that express the cre under the PR promoter. The crossing with a cre-line removes the stop codon and allows for the expression of the Ca^2+^ sensor in all tissues expressing the cre. Thus, in the offspring of these mating, the GCamp3 Ca^2+^ sensor will be present in all tissues expressing PR, amongst which the uterine epithelium.

### Cell culture

#### Mouse endometrial epithelial and stromal cells

The isolation of mouse endometrial epithelial (mEEC) and stromal (mESC) cells was performed as previously described [14]. Briefly, four to five female mice (C57Bl/6 J, Janvier France) were placed on male bedding the day before isolation to synchronize their estrous cycle. At the time of isolation, the estrous cycle phase was determined via vaginal smear examination. The mice were sacrificed whereafter the uteri were removed and placed in ice cold Hank’s Balanced Salt Solution 1X (Gibco, Thermo Fischer Scientific, Belgium) supplemented with 2% penicillin–streptomycin (Gibco, Thermo Fischer Scientific, Belgium) (HBSS +). After removal of fat and mucus, the uteri were cut open longitudinally and incubated for 1 h at 4 °C, 45 min at room temperature (RT) and 15 min at 37 °C in HBSS + supplemented with 2.5% pancreatin (Sigma-Aldrich, Belgium) and 0.25% trypsin (from bovine pancreas, Sigma Aldrich, Belgium). Thereafter, the uteri were vortexed and rinsed in HBSS + for 3 consecutive times before all solutions were pipetted onto a 100 µm cell strainer (BD Falcon, Fisher Scientific, France) in order to obtain the epithelial sheets. The cell suspension was centrifuged at 500 × g for 5 min and ultimately seeded onto the appropriate cell culture plates. After isolation of mEEC, the uteri were further digested in 0.05% Trypsin–EDTA solution (Gibco, Thermo Scientific, Belgium) supplemented with 0.1 mg/ml type IA collagenase (Sigma-Aldrich, Belgium) for 30 min on a shaker at 37 °C. After digestion, the solution was gently shaken for 10 s to release the stromal cells. The uteri were further rinsed in HBSS + , transferred to mESC growth medium and shaken. This was repeated for 3 consecutive times. Cells were finally seeded onto the desired cell cultures plates and cultured in Dulbecco modified Eagle’s medium (DMEM)/F12 (Gibco, Thermo Scientific, Belgium) supplemented with 10% fetal bovine serum (FBS), (Gibco, Thermo Scientific, Belgium), 0.5 µg/ml amphotericin B (Gibco, Thermo Scientific Belgium) and 100 µg/ml gentamicin (Gibco, Thermo Scientific, Belgium).

#### Human endometrial organoids

Human endometrial organoids of healthy women (hEMO) were cultured as described by Boretto et al. [15]. Organoids were exposed to hormonal treatments mimicking the uterine microenvironment during the menstrual cycle [15, 16]. The following protocol was used: 2 days endometrial medium (no hormonal supplement), followed by a supplement of β-estradiol (E2; 10 nM) for 2 days to induce the proliferative phase and finally 4 days of EPC (E2 (10 nM) + progesterone (P4; 1 µM) + cAMP (0.5 mM)) to induce the mid-secretory phase (including the window of implantation) [16, 17].

### RNA extraction and RT-qPCR

Total RNA was extracted from mEEC and hEMO using the RNeasy mini kit (Qiagen, The Netherlands) according to the manufacturers’ guidelines. RNA concentration and quality were evaluated using the Nanodrop method (Isogen Life Science, Belgium). cDNA was generated from 1 µg total RNA using the First-Strand cDNA Synthesis Kit (GE Healthcare, Belgium). RT-qPCR was performed using specific TaqMan gene expression assays (Life Technologies, Belgium) in the StepOne PCR system (Applied Biosystems, Belgium). Phosphoglycerate Kinase 1 (*Pgk1*) and TATA-Box Binding Protein (*Tbp*) were used as endogenous controls for the analysis of gene expression in mEEC. For the hEMO, the endogenous controls were hypoxanthine phosphoribosyl transferase 1 (*HPRT1*) and phosphoglycerate kinase 1 (*PGK1*). Data are represented as mean ± SEM of 2^(−ΔCt)^ for which ΔCt = Ct_gene of interest_ – Ct_geometric mean of endogenous control_. All experiments were performed on triplicate cDNA samples.

### RNAscope in situ hybridization

In situ hybridization (ISH) of *Par-2* (*F2rl1*), *E-cadherin* (*Cdh1*), *Trpv6* and *Enac* (*Scnn1a*) on isolated EEC, hEMO, uterine and kidney tissue were performed using the RNAscope Multiplex Fluorescent Reagent Kit (Advanced Cell Diagnostics, US). All ISH assays were carried out according to the manufacturers’ guidelines for formalin-fixed, paraffin embedded samples. Images were taken by the use of a fluorescence microscope (Nikon Eclipse Ci-E) with constant gain and exposure times at a 10 × or 20 × magnification for all images.

### Immunohistochemistry

FOXA2 immunostaining was performed on 4 µm cross-section of paraffin-embedded uterine tissue derived from 8–12 weeks old control and PUGKO animals. The sections were deparaffinized in xylene and rehydrated in graded alcohol series. Antigen retrieval was performed by boiling the uterine sections in 10 mM citrate buffer (pH 6) for 60 min. After a block with 3% normal goat serum, the sections were incubated overnight with primary rabbit monoclonal anti-FOXA2 antibody (2 µg/ml; Abcam ab108422). Afterwards, the tissues were rinsed in TRIS-buffered saline (TBS) and peroxidase-labeled secondary goat anti-rabbit antibody was used to localize FOXA2. Finally, the sections were counterstained with Maeyer Hematoxylin before mounting.

Human blastocysts (day post fertilization (dpf) 5 and dpf6) were collected at the Leuven University Fertility Centre with approval of the Ethical Committee of the UZ/KU Leuven (S62765) and the federal Commission for medical and scientific research on embryo’s in vitro, after written informed consent of the patient. Intact human blastocysts were individually fixed in 4% paraformaldehyde (PFA) for 20 min at RT. After fixation, samples were washed in 3% bovine serum albumin (BSA)/ phosphate buffered saline (PBS) and permeabilized with 0.5% Triton X-100 (T-8787, Sigma-Aldrich, USA) for 20 min at RT. The blastocysts were incubated in 10% FBS/PBS blocking solution for 30 min at RT and subsequently incubated with primary Anti-Trypsin (D-1) antibody (sc-137077) or PBT control (0.1% Triton + 3% FBS) in 10% BSA/PBS overnight at 4 °C in the dark. Blastocysts were washed in 3% BSA/PBS before incubation with secondary antibody goat anti-mouse Alexa Fluor 647 (Abcam ab150115) for 1.5 h at RT in the dark. Samples were washed in 3% BSA/PBS before incubation with HOECHST (10 nM; 33,342; Thermo Scientific) for nuclear staining. Samples were again washed in 3% BSA/PBS and finally mounted on glass bottom dishes (Cellvis). Confocal scanning microscopy was used to obtain fluorescent images. A Nikon Ti2 inverted AX R microscope was used in combination with a 20 × Plan Apo VC objective lens. The setup was controlled by NIS-Elements (NIS 5.40, Nikon Instruments Europe). DAPI and far red were respectively excited with 405 and 647 nm and the emission was collected with 429-474 nm and 662-737 nm filters. For post processing, NIS-Elements (5.40, Nikon Instruments Europe) was used. The intensity of the z-stacks was equalized (in z) via histogram stretching and the images were denoised using denoise.ai.

### RNA sequencing

#### Bulk RNA sequencing

Three independent isolations were used for these experiments. After mEEC were isolated according to abovementioned protocol, cells were incubated with trypsin (2 µg/ml) or control medium and collected at 0 h (baseline), 12 h and 24 h after incubation. Cells were collected in lysis buffer (RNeasy mini kit, Qiagen) and stored at -80 °C until further processing. Total RNA was extracted using the RNeasy mini kit (Qiagen, The Netherlands), according to the manufacturers protocol. The quality was evaluated using the Nanodrop method (Isogen Life Science, Belgium). The sequencing libraries were prepared with the Illumina TruSeq Stranded mRNA sample preparation protocol according to the manufacturers’ guidelines and sequenced on an Illumina NovaSeq6000 at the Nucleomics Core, VIB, Belgium. The reads were aligned to the GRCm38 mouse genome assembly (GENCODE release M25 [18]) using version 2.7.1 of the STAR software [19], and the transcript abundance was quantified as TPM (transcripts per million) with RSEM 1.3.1 [20].

#### Single cell sequencing

Isolated mEEC from control and PUGKO samples were mechanically processed to obtain single cells and resuspended in 0.04% BSA in PBS. Library preparation was performed using the Chromium-based single cell 10X Genomics platform according to the manufacturers protocol (10 × Genomics, single cell RNA sequencing 3′, Chromium v2). Afterwards, samples were sequenced on an Illumina HiSeq (mean reads per cell: ~ 100.000—125.000) and processed using the Cell Ranger pipeline (10X Genomics) and Seurat program for single cell genomics (Satijalab).

Single cell sequencing data of the human endometrium were obtained via publicly available servers at www.reproductivecellatlas.orgwww.reproductivecellatlas.org. Gene expression was evaluated within the Seurat program for single cell genomics (Satijalab).

Mouse endometrial single cell sequencing data were obtained via publicly available servers at Gene Expression Omnibus (GEO). Gene expression was evaluated within the Seurat program for single cell genomics (Satijalab).

### Functional measurements

#### Pharmacology

Trypsin (from porcine pancreas; 2 µg/ml), elastase (from porcine pancreas; 3U/ml), aprotinin (20 µg/ml), amiloride (10 µM) and nifedipine (10 µM) were purchased from Sigma-Aldrich, Belgium. The selective PAR2 activator, 2-furoyl-LIGRLO-NH_2_ (5 µM), store operated calcium entry (SOCE) inhibitor YM58483 (1 µM) and Phospholipase C (PLC) inhibitor U73122 (5 µM) with its negative control U73343 (5 µM) were obtained from Tocris, Bioscience, UK. The specific PAR2 inhibitor I-191 (100 nM) was purchased at Axon Medchem, The Netherlands. Ionomycin (2 µM) was used as a positive control at the end of each experiment. All stock solutions were prepared in Milli Q water, DMSO or EtOH according to the manufacturers’ guidelines.

#### Calcium microfluorimetry

The calcium measurements were performed as previously described [21]. Absolute calcium concentrations were calculated from the ratio of the fluorescence signals at both wavelengths (F340/F380) after correction for the individual background fluorescence signals, using the Grynkiewicz Equation [22]:

$$\left[{\mathrm{Ca}}^{2+ }\right]= {\mathrm{K}}_{\mathrm{eff}} \frac{\mathrm{R}- {\mathrm{R}}_{0}}{{\mathrm{R}}_{1}-\mathrm{R}}$$

for which the calibration constants R_0_, R_1_ and K_eff_ were determined as followed: R_0_ defines the ratio in Ca^2+^ free medium supplemented with 10 mM EGTA, whereas R_1_ comprises the ratio in high Ca^2+^ medium (10 mM). K_eff_, the effective binding constant, includes R_0_, R_1_, the dissociation constant of indicator dye K_D_, and the isocoefficient α, according to the following equation:

$${\mathrm{K}}_{\mathrm{eff}}= {\mathrm{K}}_{\mathrm{D}} \frac{{\mathrm{R}}_{1}+ \mathrm{\alpha }}{{\mathrm{R}}_{0}+ \mathrm{\alpha }}$$

The K_D_ of Fura-2 and the isocoefficient α were assumed as described by Zhou and Neher [23]. Cells were considered responders if the amplitude of the rise in intracellular calcium during agonist application exceeded 100 nM and when the highest value of the derivative of the calcium trace during the application of an activator exceeded at least 3 times the standard deviation (SD) of the derivative during basal conditions. Oscillating cells were defined as cells showing at least 2 peaks of intracellular calcium increase, with at least one peak exceeding 100 nM and/or cells displaying a visually obvious oscillatory pattern (≥ 4 peaks). Frequency was calculated based on the number of calcium peaks divided by the time of stimulus application. Only cells that responded to the positive control, ionomycin, at the end of the experiment were taken into account. The following bath solution was used for all measurements (in mM): 150 NaCl, 2 CaCl_2_, 1 MgCl_2_, 10 D-glucose, 10 HEPES, pH 7.4 with NaOH. In calcium-free conditions, CaCl_2_ was omitted, MgCl_2_ increased to 2.5 mM, and the solution was supplemented with 5 mM EDTA. In sodium-free conditions, NaCl was replaced with 150 mM CsCl. All data points originate from at least three independent experiments (n ≥ 3).

#### Ex vivo calcium imaging

Female C57Bl/6 J mice between 8–12 weeks old were placed on male bedding the day before the experiment to synchronize their cycle phase. Prior to sacrifice, the cycle phase was determined via vaginal smear cytology [24]. Animals were sacrificed and uterus collected in cold (4 °C) synthetical interstitial fluid (SIF) solution. The SIF solution contained (in mM): 125 NaCl, 26.2 NaHCO_3_, 1.67 NaH_2_PO_4_, 3.48 KCl, 0.69 MgSO_4_, 9.64 D-gluconic acid, 5.55 D-glucose, 7.6 Sucrose and 2 CaCl_2._ The pH was buffered with carbogen gas to pH 7.4. The collected uteri were opened longitudinally and each horn was cut in half. Next, the tissue fragment was incubated in SIF solution at 4 °C to rest for at least one hour. For image acquisition, tissue fragments were fixed with the inner uterus lining towards the objective in a glass-bottom microwell dish (MatTek, 35 mm petri dish, Ashland, USA) and incubated in SIF solution supplemented with the chemical compound of interest. Image acquisition was performed using a Nikon NiE microscope equipped with a Yokogawa CSU-X spinning-disk module with dual camera (Andor iXon3) in combination with a Plan Fluor 10 × air objective (NA 0.30, 16 WD). The images were adjusted for movement using Fiji ImageJ (Linear Stack Alignment with SIFT plugin) and regions of interest were selected. Raw fluorescent traces were extracted and converted to ΔF/F_0,_ for which the fluorescence at any given point is represented by F, and F_0_ is the background fluorescence.

#### Whole-cell path clamp experiments

Patch clamp recordings were measured with an EPC-10 amplifier and PatchMasterPro Software (HEKA Elektronik, Lambrecht, Germany). Current measurements were performed at a sampling rate of 20 kHz and currents were digitally filtered at 2.9 kHz. The pipette solution contained (in mM): 100 CsAsp, 45 CsCl, 10 EGTA, 10 HEPES, 1 MgCl_2_ (pH 7.2 with CsOH). The extracellular solution contained (in mM): 150 NaCl, 10 HEPES, 10 Glucose, 2 CaCl_2_, 1 MgCl_2_, (pH 7.4 with NaOH). The standard patch pipette resistance was between 2 MΩ and 4 MΩ when filled with pipette solution. 50–70% of the series resistance was compensated during the recordings. The applied voltage step protocols are described in the corresponding figure legends.

### Data analysis and statistics

Calcium imaging and electrophysiological data were analyzed using IgorPro 6.2 (WaveMetrics, USA), WinASCD (Guy Droogmans, Leuven, Belgium) and OriginPro 8.6 (OriginLab Corporation, USA). RNA seq data was analysed in RStudio 1.4.1717 (RStudio, PBC). OriginPro 8.6 was further used for data display. Statistics were performed using GraphPad Prism 8.4.3 for Windows (GraphPad Software, USA). Statistical significance was considered when *p* < 0.05.

## Results

### Serine protease trypsin induces intracellular calcium oscillations in mouse endometrial epithelial cells

To start, the effect of continuous release of proteases by the blastocyst on the EEC was evaluated. To validate the luminal origin of mouse EEC (mEEC), a primary culture was initiated and compared to mEEC derived from the progesterone-induced uterine gland knock out (PUGKO) mouse model [25]. The lack of uterine glands in PUGKO mice was confirmed by immunohistochemical analysis and RT-qPCR data (Supp. Figure 1A, B). Single cell sequencing analysis of PUGKO-derived mEEC illustrated no expression of *Foxa2*, a typical glandular epithelial cell marker (Supp. Figure 1I, J). In contrast, *Foxa2* could be detected in mEEC derived from control animals. Notably, this *Foxa2* positive cluster was rather small, indicating the overall luminal character of the isolated epithelial cells (Supp. Figure 1E, F).

**Fig. 1:**
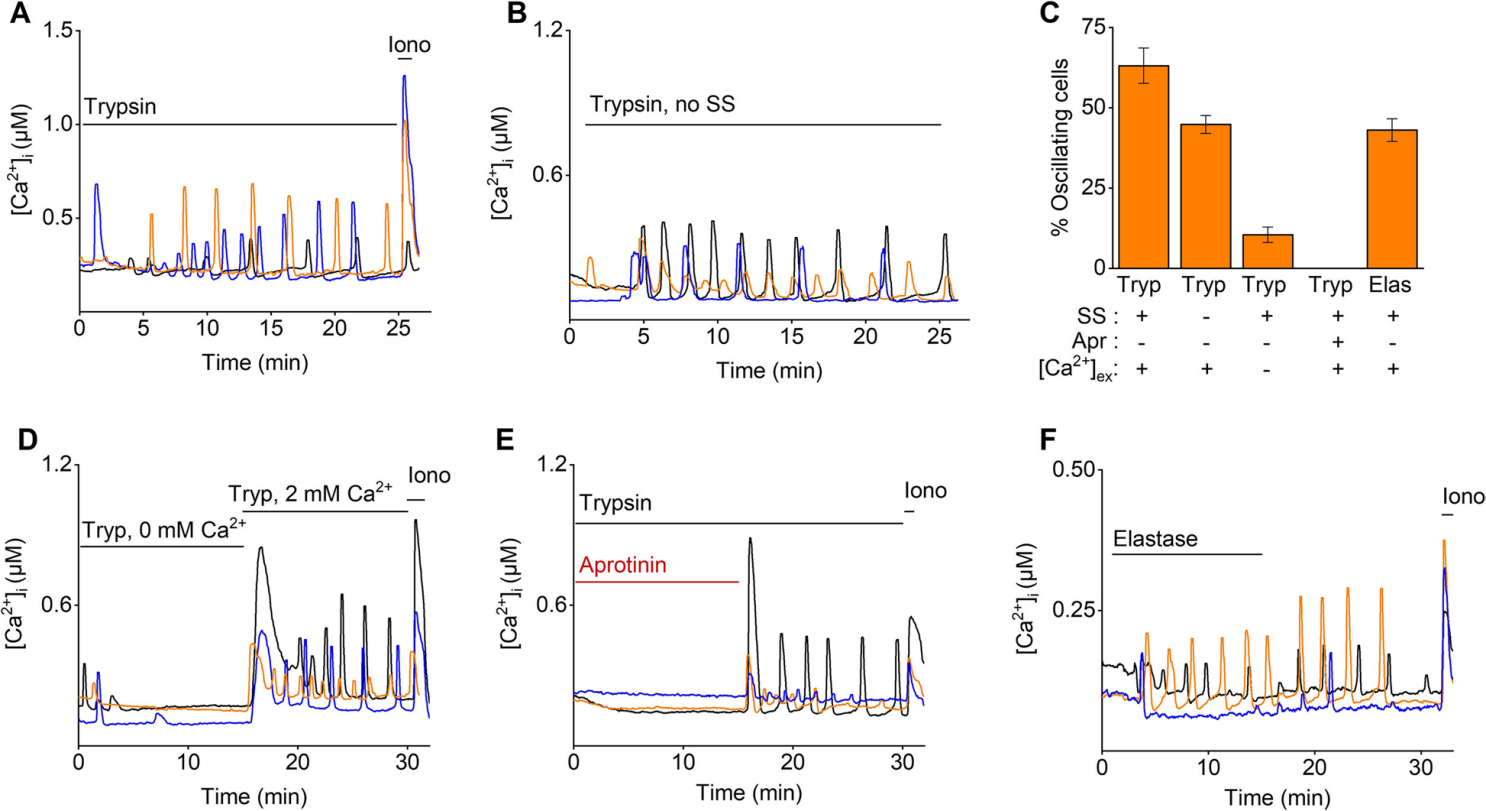
Trypsin induces calcium oscillations in mouse endometrial epithelial cells. **A** Representative traces of mEEC stimulated with trypsin (2 µg/ml) in the presence or **B** absence of shear stress (SS) **C** Percentage of oscillating cells (≥ 2 intracellular Ca^2+^ peaks). The presence ( +) or absence (-) of Aprotinin, shear stress and extracellular Ca^2+^ is shown. Data are visualized as mean ± SEM. **D** depicts trypsin responses in the presence (2 mM Ca^2+^) or absence (0 mM Ca^2+^) of extracellular Ca^2+^. In **E** trypsin responses were challenged with aprotinin (10 µg/ml). **F** Representative Ca^2+^ traces of mEEC upon stimulation with elastase (3U/ml). Ionomycin (2 µM) was added at the end of each experiment as a positive control. *n* = at least 3 independent experiments with a total minimum of 200 cells per condition. Iono = ionomycin, Tryp = trypsin, Apr = aprotinin, SS = shear stress

Next, [Ca^2+^]_I_ of control mEEC was monitored using Ca^2+^ microfluorimetric experiments. The onset of perfusion induced a small laminar flow of 307 µl/min over the cells and mimicked shear stress (SS). Intriguingly, application of SS alone evoked [Ca^2+^]_I_ oscillations in a significant portion of mEEC (22 ± 2%; Supp. Figure 2A, D), which were absent when perfusion, and thus shear stress was not applied (Supp. Figure 2B). These findings argue against the presence of spontaneous [Ca^2+^]_I_ oscillations but indicate that shear stress induced by the perfusion system is sufficient to evoke them. Subsequently, mEEC were stimulated with the serine protease, trypsin (2 µg/ml), for a time period of minimally 25 min, which resulted in [Ca^2+^]_I_ oscillations in the majority of mEEC (63 ± 5%) (Fig. 1A and C). These data were comparable to the recently described results in Ishikawa cells [13]. A similar trypsin stimulation in the absence of SS resulted in [Ca^2+^]_I_ oscillations in mEEC, albeit in a lower number of cells (44 ± 3%) (Fig. 1B, C). In the absence of extracellular Ca^2+^, the observed oscillating [Ca^2+^]_I_ pattern was strongly reduced and quenched in function of time (17 ± 2% oscillating mEEC) (Fig. 1C and D). Reapplication of external Ca^2+^ restored the oscillatory pattern, indicating that extracellular Ca^2+^ entry is required to trigger trypsin-induced [Ca^2+^]_I_ oscillations. Moreover, the trypsin-induced [Ca^2+^]_I_ oscillations were completely abolished in the presence of aprotinin, a serine protease inhibitor (Fig. 1C and E). Additionally, elastase, another member of the serine protease family, also induced [Ca^2+^]_I_ oscillations in mEEC (43 ± 4%) in the presence of SS, with an oscillation frequency similar to application of trypsin (0.26 ± 0.05 and 0.27 ± 0.02 oscillations/min for trypsin and elastase respectively) (Fig. 1C, F). Taken together, these results confirmed that serine proteases, such as trypsin and elastase, induce [Ca^2+^]_I_ oscillations in luminal mEEC.

### Mouse EEC do not express voltage-dependent Ca^2+^ channels at the functional level

**Fig. 2:**
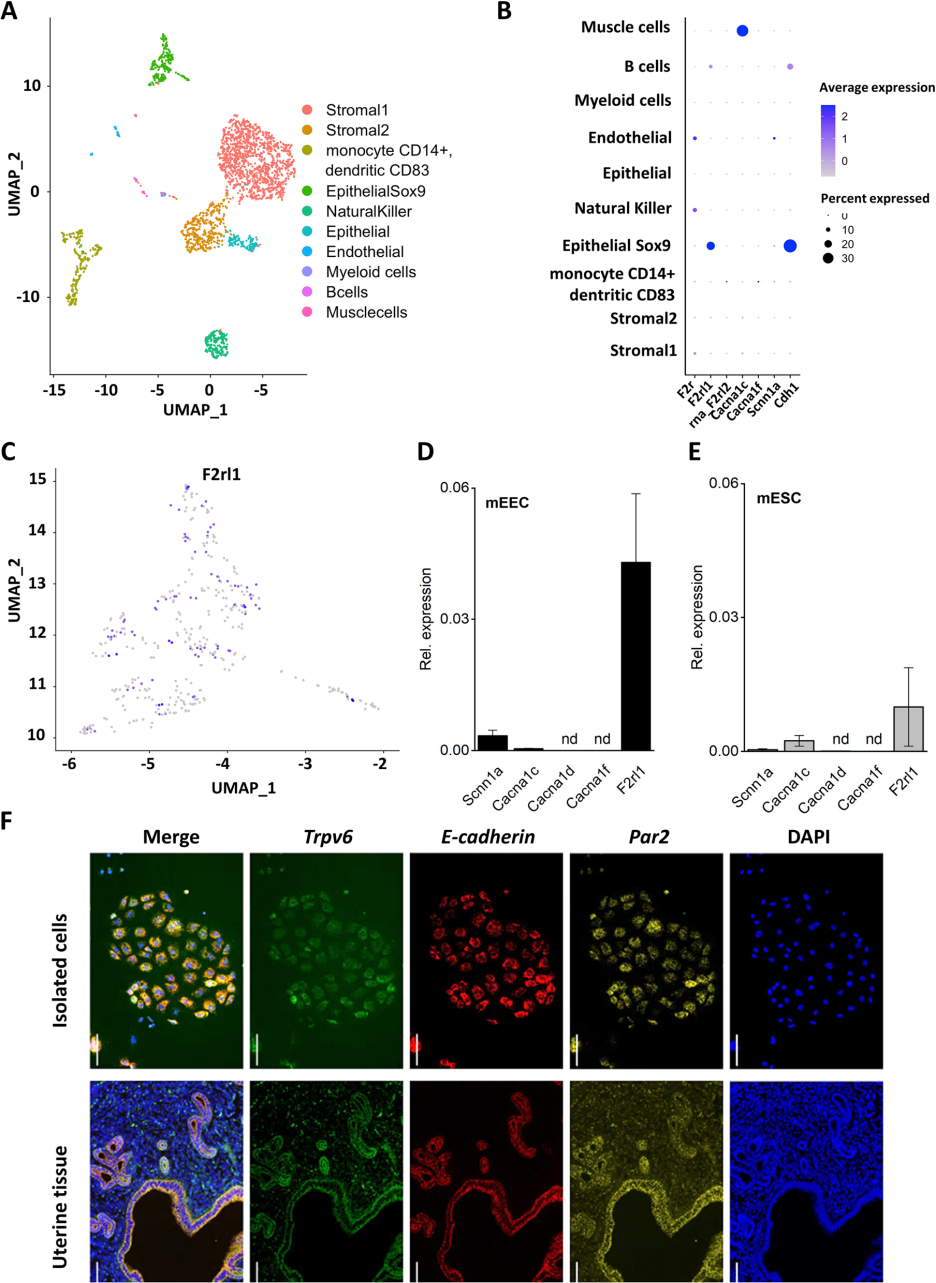
Expression of PAR2, ENaC and VDCC in mEEC. **A** Single cells RNA sequencing data on two different mouse endometrial cells cultures obtained from public datasets [26]. Uniform manifold approximation and projection of cell populations identified in the merged endometrial mouse datasets. **B** Dot plot representing the gene counts in the merged datasets for the genes *F2r* (*Par1*), *F2rl1* (*Par2*), *F2rl2* (*Par3*), *Cacna1c* (*Ca*_*v*_*1.2*), *Cacna1f* (*Ca*_*v*_* 1.4*), *Scnn1a* (*ENaC*) and *Cdh1* (*E-cadherin*). **C** Expression of *Par2* in Uniform manifold approximation and projection (UMAP) space of the subpopulation mouse endometrial epithelial Sox9 positive cells are shown. **D**, **E** mRNA expression of *Scnn1a, Cacnca1c, Cacn1d,cacna1f and F2rl1* in isolated mEEC (**D**) and mESC (**E**). Expression is relatively quantified compared to geometric mean of the housekeeping genes Gapdh. (Data is shown as mean ± SEM. *N* = 3.) **F** In situ hybridization RNAscope images of isolated mEEC (left panels) and uterine tissue sections (right panels). Positive signals were detected for *Par2* and the epithelial markers *E-cadherin* and *Trpv6.* Scale bar: 50 µm

Next, we aimed to unravel the downstream signaling pathway involved in [Ca^2+^]_I_ oscillations induced by serine proteases in mEEC. Previous reports described a potential role of the epithelial sodium channel (ENaC) and the voltage dependent Ca^2+^ channel (VDCC) in [Ca^2+^]_I_ responses to embryo-derived proteases [12]. Evaluation of the public datasets GSM2906479 and GSM2906478 confirmed modest mRNA levels of *Scnn1a* (ENAC) and absence of *Slc8a1* (sodium calcium exchanger (NCX)) (Fig. 2A, B). However, only mRNA levels of a single subunit of VDCC *Cacna1c* (Ca_v_ 1.2) could be detected while expression levels of *Cacna1c* (Ca_v_ 1.2), *Cacna1d* (Ca_v_ 1.3) and *Cacna1f* (Ca_v_ 1.4) were below detection levels in mEEC (Fig. 2B). These results were further confirmed using RT-qPCR on primary isolated mEEC (Fig. 2D). Via RNAscope in situ hybridization, only a modest signal for *Scnn1a* (ENaC) was detected in uterine horn tissue sections (Supp. Figure 3A), with the specificity of the *Scnn1a* probe validated on kidney tissue as a positive control (Supp. Figure 3B).

**Fig. 3:**
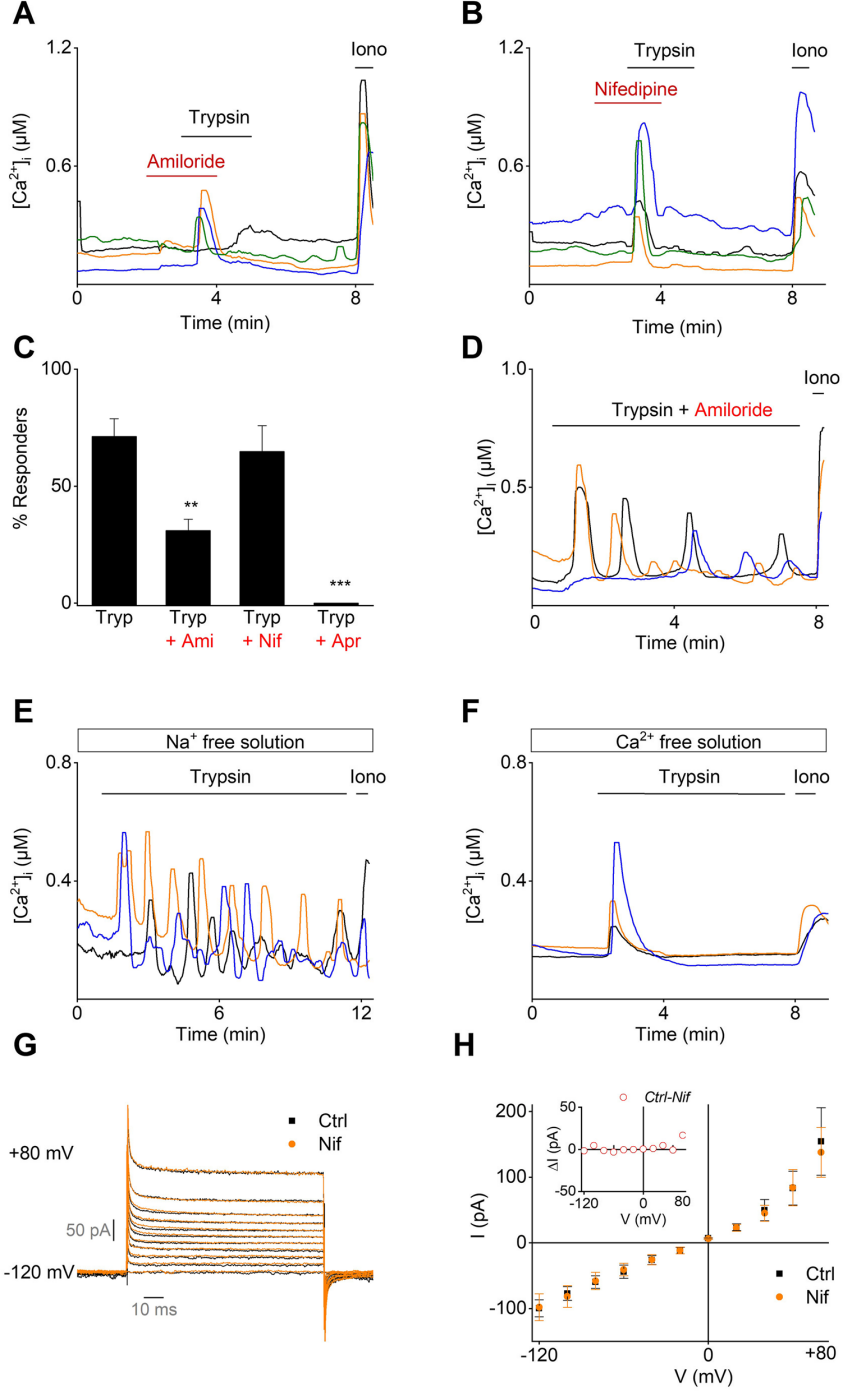
No role of ENaC and VDCC in the Ca^2+^ response. **A**-**B** Ca^2+^ microfluorimetry. Representative traces of mEEC stimulated with trypsin (20 µg/ml) challenged with (**A**) amiloride (10 µM), **B** nifedipine (10 µM). **C** Displays the percentage of responding cells to stimulation with either amiloride (Ami), nifedipine (Nif) and aprotinin (Apr, (10 µg/ml). Data is represented as mean ± SEM. ** *p* < 0.01, using one way ANOVA corrected from multiple testing with Dunnett’s multiple comparison test and compared to the trypsin condition. **D** Representative traces of mEEC stimulated with amiloride (10 µM) + trypsin (20 µg/ml). **E**–**F** Representative traces for trypsin stimulation of primary mEEC in Na^+^-free and Ca^2+^-free extracellular solution. Ionomycin (2 µM) was applied at the end of each protocol as positive control. *N* = at least 3 different experiments on a total minimum of 300 cells. **G** Whole-cell patch-clamp experiments of mEEC applying a voltage step protocol ranging from -120 mV holding to + 80 mV in + 20 mV steps in control (Ctrl) condition and in the presence of nifedipine (3 µM), an inhibitor of VDCC. **H** Current (*I*) Voltage (*V*) relationship for currents extracted from steady-state currents in. Insert represents the difference in current amplitude between control (Ctrl) and nifedipine (Nif) condition. Tryp = trypsin, Am = amiloride, Nif = nifedipine, Apr = aprotinin, Iono = ionomycin

Next, the trypsin-induced membrane depolarization and the subsequent activation of VDCC in mEEC was further investigated by Ca^2+^ microfluorimetric experiments. Remarkably, the [Ca^2+^]_I_ influx was only partially reduced by the simultaneous presence of trypsin with either amiloride or nifedipine, inhibitors of ENaC and VDCC, respectively (Fig. 3A - C). In addition, the trypsin-induced [Ca^2+^]_I_ oscillations were not affected in the presence of amiloride (Fig. 3D), or when Na^+^ was omitted from the external bath solution. However, trypsin-induced [Ca^2+^]_I_ oscillations were completely absent in a Ca^2+^-free extracellular environment (Fig. 3F). Moreover, whole-cell patch clamp experiments applying a specific voltage-step protocol to activate VDCC did not provide any evidence for the functional expression of VDCC in mEEC (Fig. 3G and H). Altogether, these data suggest that trypsin-induced [Ca^2+^]_I_ influx is independent of the activity of ENaC and VDCC in mEEC.

### PAR2 is involved in [Ca^2+^]I oscillations in mEEC which are dependent on the PLC/IP_3_R pathway and STIM1/Orai1-interaction

Other proposed molecular players to be involved in the transduction of embryonic signals are members of the family of protease-activated receptors (PARs) [27]. RNA sequencing data from two publicly available mouse uterus datasets [26] showed a high gene count for *F2rl1* (mEEC Sox 9; Fig. 2A-C), the gene that encodes for PAR2, whereas only low expression could be detected for *F2r,* corresponding to PAR1, and no expression could be detected for *F2rl2* and *F2rl3,* PAR3 and PAR4 respectively, in the epithelial cells (Fig. 2B). The epithelial expression of *F2rl1* (PAR2) was further confirmed via RNAscope in situ hybridization, which revealed prominent positive staining for *Par2* in isolated mEEC as well as tissue sections of the intact uterine horn (Fig. 2F). The expression of *Cdh1 (*E-cadherin) and *Trpv6* were included as positive controls for endometrial epithelial cells. The merged images showed prominent co-expression of *Par2* with E-cadherin and *Trpv6* (Fig. 2F). Ultimately, similar results were obtained using RT-qPCR, validating the molecular presence of F2rl1 (PAR2) in mEEC (Fig. 2D).

**Fig. 4:**
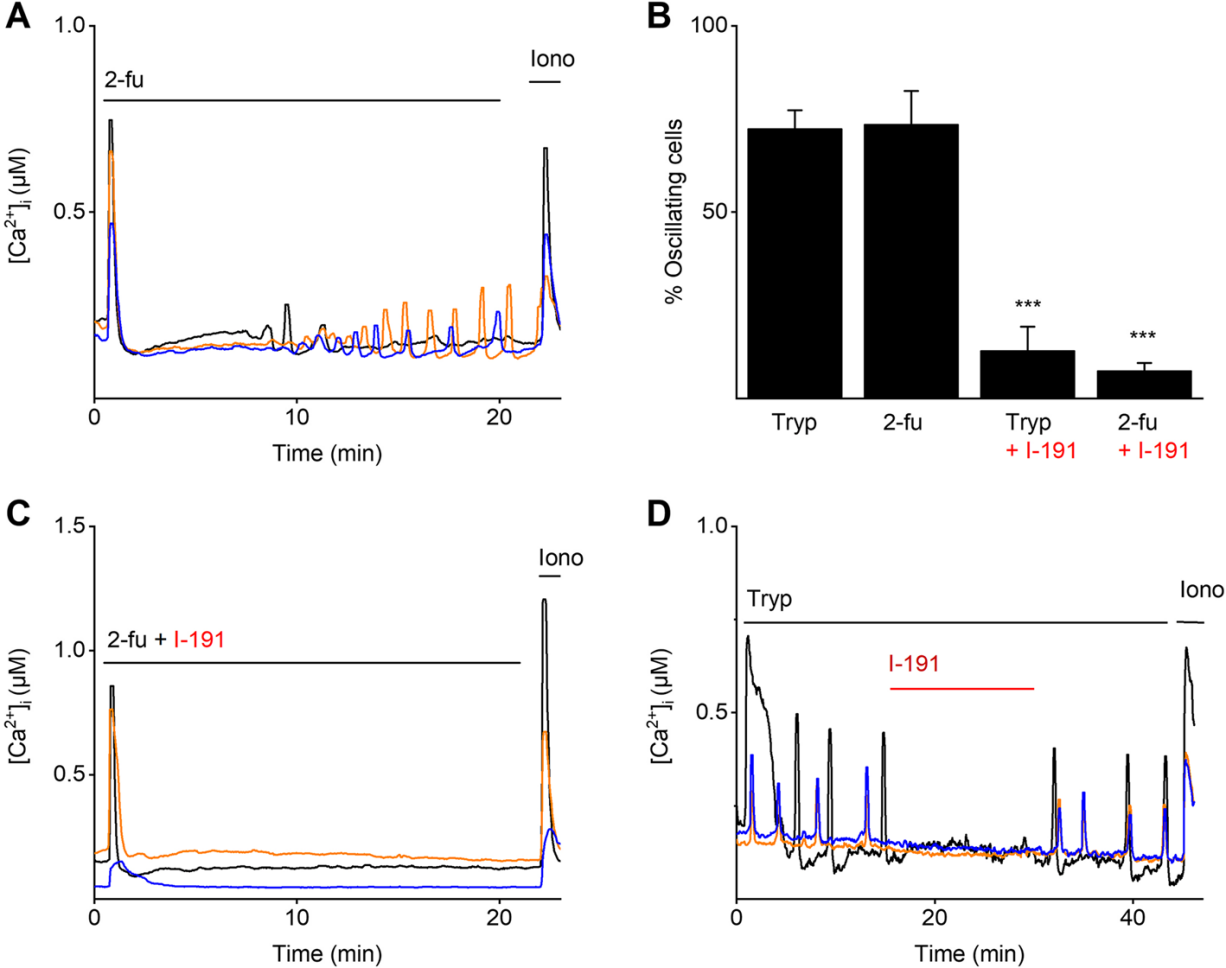
Validation of the functional PAR2 expression in mEEC. Ca^2+^ microfluorimetry. **A** Representative traces of 2-furoyl-LIGRLO-NH_2_ (5 µM), the PAR2 agonist, stimulation of mEEC. **B** Percentage of oscillating cells in the responding cell population to trypsin (2 µg/ml), 2-furoyl-LIGRLO-NH_2_ (5 µM) and the simultaneous application of either trypsin or 2-furoyl-LIGRLO-NH_2_ with the specific PAR2 inhibitor I-191 (100 nM). Data is shown as mean ± SEM. *** *p* < 0.001 compared to the trypsin application. Statistical significance was analyzed with a one-way ANOVA corrected for multiple comparisons with Dunnett’s multiple comparison test. **C**-**D** Trypsin or 2-furoyl-LIGRLO-NH_2_ responses were challenged with the inhibitor of PAR2, I-191 (100 nM). Ionomycin was added at the end of each experiment as a positive control. *N* = at least 3 different experiments with a total minimum of 300 cells

Next, functional expression of PAR2 in mEEC was assessed using Ca^2+^ microfluorimetric experiments. Application of 2-furoyl-LIGRLO-NH_2_ (2-fu), a selective PAR2 agonist [28], induced [Ca^2+^]_I_ oscillations in mEEC. Typically, a rapid first [Ca^2+^]_I_ peak was detected, followed by [Ca^2+^]_I_ oscillations (Fig. 4A). The percentage of oscillating cells upon 2-fu application was comparable to the percentage of oscillating cells upon trypsin application (72 ± 5% vs. 73 ± 9%, respectively) (Fig. 4B). Further, incubation with the PAR2 agonist 2-fu, without the influence of shear stress by the perfusion system, triggered similar [Ca^2+^]_I_ oscillations in mEEC (Supp. Figure 2C and D). Interestingly, both trypsin- and 2-fu-induced [Ca^2+^]_I_ oscillations were significantly reduced by co-application of a specific PAR2 antagonist, I-191 [29] (Fig. 4B and C) and the trypsin-induced oscillatory response was restored after wash-out of I-191 to similar levels as before (Fig. 4D). In addition, 2-fu-induced [Ca^2+^]_I_ oscillations were unaffected by aprotinin (Supp. Figure 4).

**Fig. 5:**
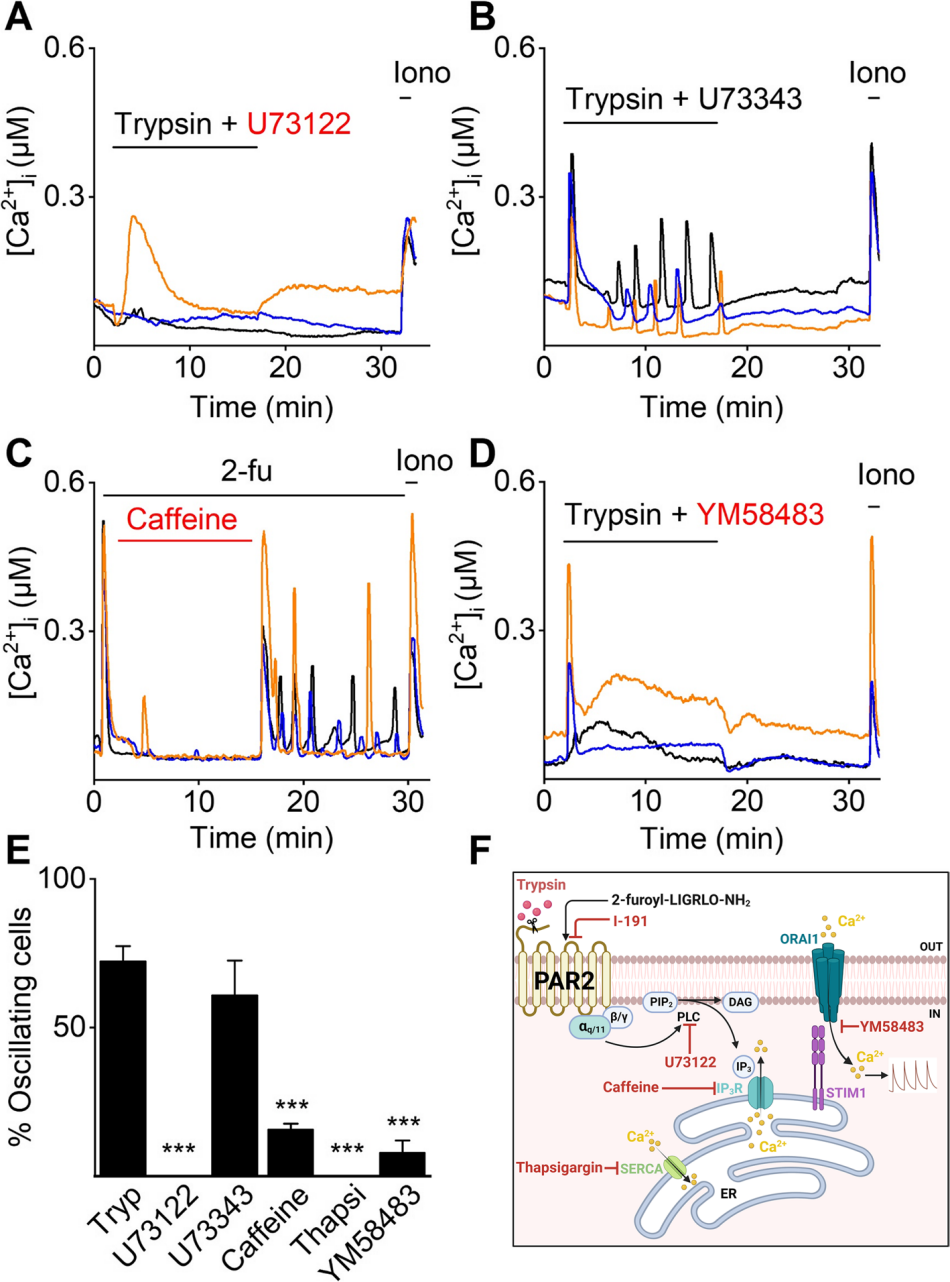
Calcium oscillations are dependent on PLC pathway and CRAC channels. Ca^2+^ microfluorimetry. Representative traces of simultaneous stimulation of mEEC with trypsin (2 µg/ml) and either the PLC inhibitor U73122 (5 µM) (**A**), the PLC negative control compound U73343 (5 µM) (**B**), the IP_3_R inhibitor Caffeine (**C**) and the CRAC inhibitor YM58483 (10 µM) (**D**). Ionomycin (2 µM) was added as a positive control. In **E** the percentage of oscillating cells within the responding cell population is shown for mEEC stimulation with trypsin (2 µg/ml), U73122 (5 µM), U73343 (5 µM), caffeine (60 mM), thapsigargin (10 µM), and YM58483 (10 µM). Experiments with thapsigargin were carried out in 0 mM extracellular Ca^2+^. Data is shown as mean ± SEM. *** *p* < 0.001 compared to the trypsin condition, using one-way ANOVA corrected for multiple comparisons with Holm-Sidak. For the PLC inhibitor and thapsigargin data, a two-sample t-test was performed. *N* = at least 3 independent experiments with a total minimum of 100 cells. Iono = ionomycin, tryp = trypsin, Thapsi = thapsigargin. **F** Schematic overview of PAR2 activated pathways and it’s modulator (figure created with BioRender.com)

The involvement of PAR2 in [Ca^2+^]_I_ oscillations was further assessed by the use of specific pharmacological tools to interfere with downstream signaling pathways. PAR2 activation initiates the dissociation of G_α_ and G_βγ_ subunits and the activated G_α_ subunit induces further downstream signals such as Ca^2+^ release from intracellular stores via the PLC/IP_3_ pathway (Fig. 5F) [27, 30]. To evaluate the participation of the downstream PLC pathway, mEECs were co-stimulated with trypsin and the PLC inhibitor, U73122. This protocol resulted in a full inhibition of the induced [Ca^2+^]_I_ oscillations, which was not observed during co-application of trypsin and the negative control, U73343 (Fig. 5B and E). Moreover, inhibition of IP_3_R via application of caffeine, a non-selective IP_3_R inhibitor [31], significantly reduced the proportion of 2-fu-induced oscillating cells (Fig. 5C and E). To assess the potential involvement of the endoplasmic reticulum (ER) in [Ca^2+^]_I_ oscillations, Ca^2+^ stores of the ER were first depleted by thapsigargin, a non-competitive inhibitor of the sarco/endoplasmic reticulum Ca^2+^ ATPase (SERCA). Interestingly, application of thapsigargin prior to stimulation of trypsin prevented the cells from responding to trypsin completely (Fig. 5E). In addition, stimulation of mEEC with a blocker of Ca^2+^ release-activated calcium channels Orai1, YM58483, prevented the onset of [Ca^2+^]_I_ oscillations whereas the initial calcium response remained intact (Fig. 5D and E), demonstrating the involvement of the store-operated calcium influx via Stim1/Orai1. In conclusion, these experiments provide evidence for the functional role of PAR2 in [Ca^2+^]_I_ oscillations in mEEC, in which the depletion of intracellular Ca^2+^ stores occurs via PLC, IP_3_R and Calcium Release Activated Channel (CRAC) channels as part of the downstream signaling.

### No functional PAR2 expression in endometrial stromal cells

Next, the expression of PAR2 was investigated in the endometrial stromal cells of mouse (mESC). Remarkably, only marginal mRNA expression levels of *F2rl1* were detected in mESC via single cell RNAseq (Fig. 2E). RNAscope in situ hybridization experiments showed that fluorescence of the *F2rl1* probe in intact uterine tissue was less pronounced in the stromal part of the uterus compared to the luminal epithelial cells in (Fig. 2F). In addition, stimulation of mESC by trypsin via the perfusion system (+ SS) did not result in [Ca^2+^]_I_ oscillations, further indicating that trypsin-induced [Ca^2+^]_I_ oscillations are specific for mEEC (Supp. Figure 5A, B).

### PAR2 is involved in [Ca^2+^]_I_ oscillations in intact mouse endometrium

The effects of the serine protease trypsin and the selective PAR2 agonist 2-fu were further evaluated via ex vivo Ca^2+^ imaging experiments on excised uterine tissues from non-pregnant mice. The uterine tissue fragments of the conditional PR-GCamp3 mice were separately incubated with either trypsin (Suppl. Movie 1), 2-fu (Suppl. Movie 2) or the standard bath solution (Suppl. Movie 3), during which [Ca^2+^]_I_ responses were visualized. Interestingly, these ex vivo experiments revealed [Ca^2+^]_I_ oscillations in whole uterine tissues upon application of SS. Both application of trypsin or 2-fu to the uterine horns significantly increased the number of [Ca^2+^]_I_ oscillations in the intact uterine wall (Supp. Figure 6). However, when Ca^2+^ was omitted from the extracellular solution, the oscillatory response was strongly reduced (Supp. Movie 4). Altogether, these data further illustrate the functional expression of PAR2 in mouse luminal epithelial cells and its involvement in triggering [Ca^2+^]_I_ oscillations in vivo.

### Serine protease trypsin induces [Ca^2+^]_I_ oscillations in human endometrial organoids

**Fig. 6:**
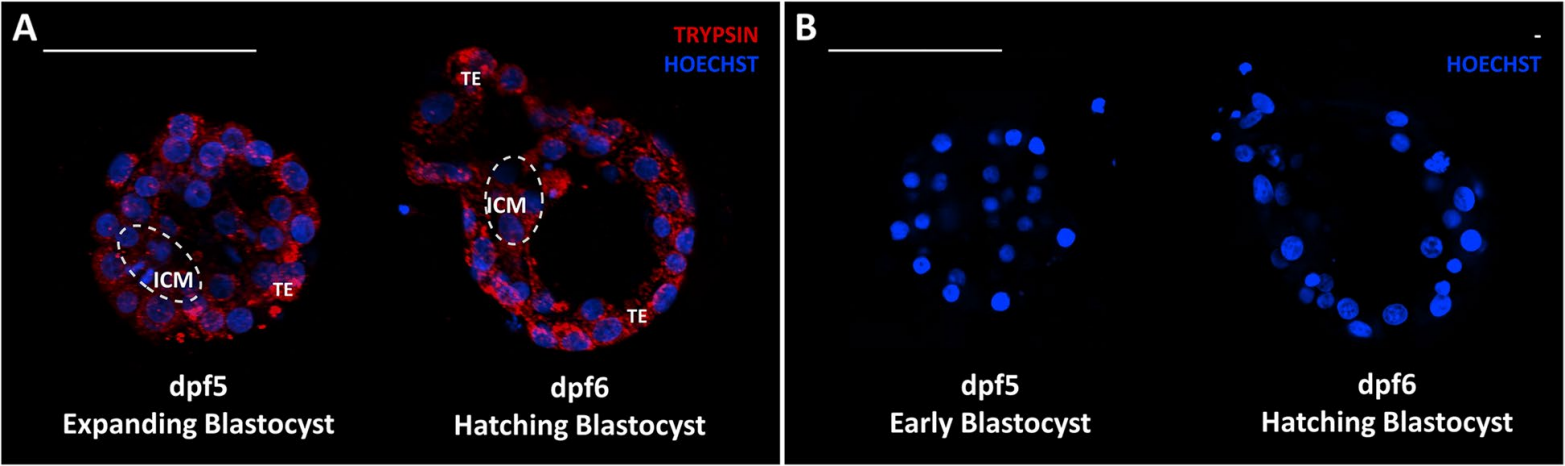
Immunofluorescent staining of human blastocysts 5 and 6 days post fertilization. **A** Trypsin immunostaining on intact human blastocysts with primary Anti-Trypsin (D-1) antibody (sc-137077). **B** Blastocysts were stained with the same staining procedure but the primary antibody was omitted. Scale bar = 100 µm. TE = trophectoderm, ICM = inner cell mass

To further evaluate whether a similar fetal-maternal communication exists in human, trypsin secretion was evaluated in human blastocysts 5 to 6 days post fertilization (dpf). Interestingly, immunofluorescent staining of human blastocysts revealed a positive signal for the presence of trypsin in trophectoderm (TE) cells of both expanding and hatching blastocysts (Fig. 6).

**Fig. 7:**
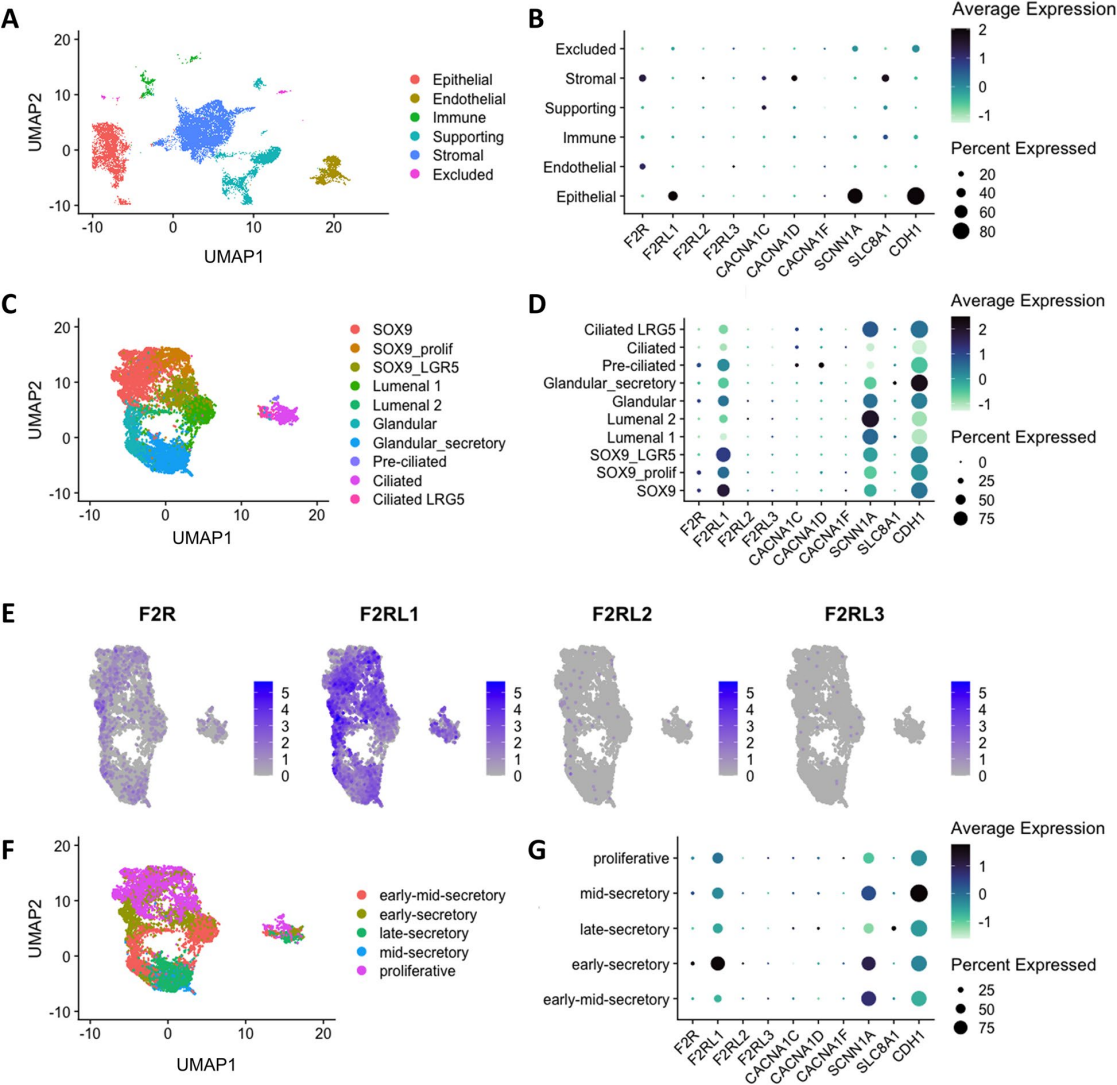
PAR2 expression in different subsets of human uterine epithelial cells. **A** Uniform manifold approximation and projection of cell populations in the human endometrium, as described by [5]. **B** Dot plot representing the average expression and expression percentages of genes in endometrium cell types. **C** UMAP of the distinct epithelial populations within the human endometrium, **D** Dot plot representing the average expression and expression percentage of several genes in the epithelial subpopulations. **E** Expression of the individual PAR-members in UMAP space. **F** UMAP representation of the epithelial subsets throughout the different stages of the menstrual cycle. **G** Dot plot showing expression levels for the genes provided in (b) in the different stages of the human cycle

Expression levels of *F2RL1* were assessed in the human endometrium by analyzing a publicly available single cell RNA sequencing dataset [5]. Expression levels of the different PAR family members (*F2R, F2RL1, F2RL2* and *F2RL3*), together with VDCC (*CACNA1C, CACNA1D* and *CACNA1F*), ENaC (*SCNN1A*) and NCX (*SLC8A1*) were investigated amongst the epithelial subsets (Fig. 7B). *F2RL1* and *SCNN1A* were most abundantly expressed in epithelial cells, whereas minimal expression was observed in stromal cells. In accordance with our findings in endometrial cells of mice, very low expression was identified for the genes encoding NCX and VDCC (*CACNA1C, CACNA1D*, and *CACNA1F*) (Fig. 7B,D and G). Interestingly, *F2RL1* and *SCNN1A* showed expression patterns in almost all epithelial subclusters (Fig. 7C and D). Among the members of the PAR family, *F2RL1* was most abundantly expressed, with the highest expression in the *SOX9-*expressing subpopulations (Fig. 7C and D). Next, the expression levels for these genes were further examined over different stages of the human cycle (Fig. 7F and G), and although *F2RL1* remained constantly expressed, epithelial cells in early secretory stage showed the highest levels.

**Fig. 8:**
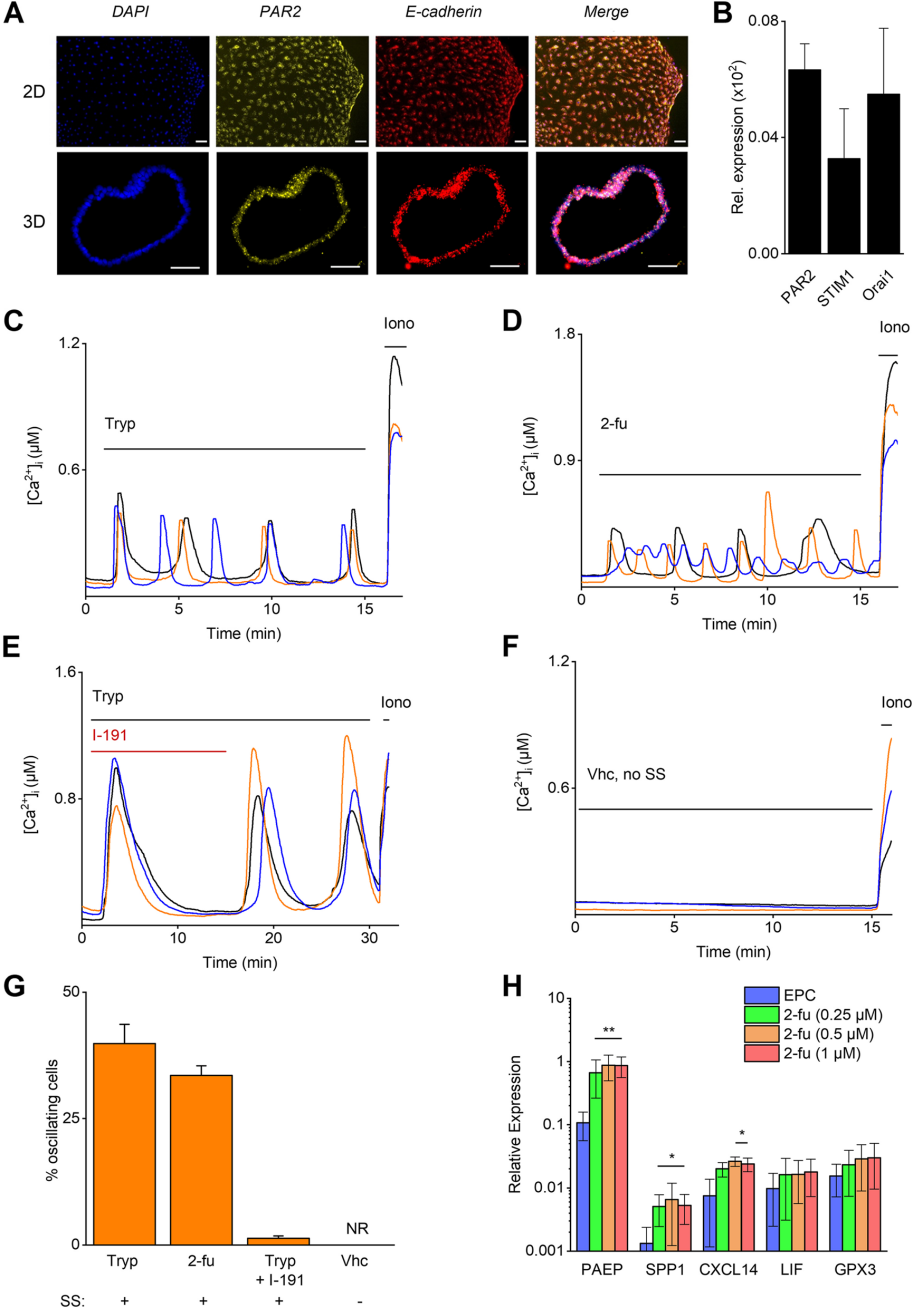
hEMO display functional PAR2 expression. **A** In situ hybridization images of hEMO in 2D and 3D conformation. Signals were detected for PAR2 (*F2rl1)* and the epithelial marker E-cadherin (*Cdh1*). Scale bar: 50 µm. **B** mRNA expression of *PAR2, ORAI1* and *STIM1* in hEMO. Expression is relatively quantified compared to geometric mean of the housekeeping genes *HPRT1* and *PGK1*. Data is shown as mean ± SEM. **C**-**G** Ca^2+^ microfluorimetry. In **C** and **D** representative traces of 2D hEMO stimulation with trypsin (1 µg/ml) or 2-furoyl-LIGRLO-NH_2_ (250 nM) are shown. In **E** trypsin responses were challenged with the specific PAR2 inhibitor I-191 (100 nM). **F** 2D hEMO treated with vehicle, without shear stress. Ionomycin (2 µM) was added at the end of each experiment as positive control. **G** Percentage of oscillating cells in response to trypsin (2 µg/ml), 2-furoyl-LIGRLO-NH_2_ (5 µM), simultaneous application of trypsin with the specific PAR2 inhibitor I-191 (100 nM) and vehicle. The presence ( +) or absence (-) of shear stress (SS) is shown. NR: no responding cells. Data is shown as mean ± SEM. *N* = at least 3 independent experiments on hEMO obtained from minimum two different patients, with a total minimum of at least 130 cells. **H** mRNA expression of decidualization markers PAEP, SPP1, CXCL14, LIF and GPX3 in hEMO. Expression is relatively quantified compared to the housekeeping gene *GAPDH*. Data is shown as mean ± SEM. *N* = 3 independent experiments. Two-way ANOVA with Dunnett’s multiple comparison test. * *p* < 0.05, ** *p* < 0.01 compared to EPC condition. Tryp = trypsin, 2-fu = 2-furoyl-LIGRLO-NH_2,_ Iono = ionomycin. SS = shear stress

PAR2 expression at the RNA level and functional expression were further examined in human endometrial epithelial organoids of healthy women (hEMO) [15, 21]. RNAscope experiments using cells in 2D culture derived from hEMO and intact 3D organoids revealed a positive signal for the presence of *F2RL1* and co-expression with the epithelial marker *CDH1* (E-cadherin) (Fig. 8A). These results were confirmed using RT-qPCR, showing prominent RNA expression of *F2RL1* (Fig. 8B). When stimulated with either trypsin or 2-fu, [Ca^2+^]_I_ oscillations were triggered in single cell-dissociated hEMO (Fig. 8C, D and G). The observed oscillatory response was similar in cells responding to trypsin and 2-fu (40 ± 4% versus 34 ± 2%). However, in the presence of the specific PAR2 inhibitor, I-191, the trypsin-induced oscillatory pattern was completely inhibited (Fig. 8E and G). As a control experiment, hEMO cells were incubated with bath solution without shear stress or trypsin and no [Ca^2+^]_I_ oscillations could be detected (Fig. 8F-G). The expression pattern of *PAR2* in the human endometrium was comparable to the mouse condition showing high expression in endometrial epithelial cells while no expression was detected in endometrial stromal cells (hESC) at the RNA level (Figs. 7B and 2E) nor at the functional level (Supp. Figure 5C, D). These data provide evidence for the involvement of PAR2 in the oscillatory [Ca^2+^]_I_ response of epithelial cells to stimulation by serine proteases in human EMO.

Finally, the physiological impact of PAR2 activation during pre-decidualization in human endometrial epithelial cells was further investigated. Therefore, hEMO were first pretreated with a hormonal supplementation protocol (E2 (10 nM) + progesterone (P4; 1 µM) + 8-Br-cAMP (0.5 mM); EPC) to obtain the secretory phase including the window of implantation and afterwards cultured with different concentrations of 2-fu (0.25 µM, 0.5 µM and 1 µM). Interestingly, RT-qPCR results showed significant upregulation of WOI- markers in the presence of the PAR2 agonist. The expression levels of *PAEP* and *SPP1* were increased compared to EPC-only treated cells, while expression of *CXCL14* was significantly elevated at 500 nM and 1 µM (Fig. 8H). In summary, these data show a relevant biological impact of PAR2 activation on pre-decidualization events.

## Discussion

The communication and complex interplay between the implanting blastocyst and the maternal endometrium are imperative during the embryo implantation process. The molecular players involved in both embryonic signal generation and maternal signal detection have been extensively studied, but a full comprehension of the mechanisms at play is still lacking. Especially the identification of molecular signals governing embryo-uterine communication, and their function in human endometrial epithelial cells remain incomplete. Recently, serine proteases have been acknowledged as blastocyst-secreted proteins that are able to act on the maternal endometrium and influence the implantation process [8, 12]. However, the molecular response by the maternal endometrium is currently not characterized properly. In this study, we showed that continuous application of serine proteases like trypsin or elastase induced [Ca^2+^]_I_ oscillations specifically in EEC, but not in the underlying ESC of both mouse and human origin. Moreover, PAR2 was found to be involved in the translation of blastocyst-secreted proteases like trypsin into [Ca^2+^]_I_ oscillations, a signaling cascade which depends on the PLC/IP_3_/SOCE pathway. In addition, the presence of trypsin in the trophectoderm of (hatching) human blastocysts was further confirmed via immune histological staining techniques. These results reveal a novel role for PAR2 in the epithelium-specific reaction to embryonic signals.

Previous reports have suggested several molecular mechanisms to explain the increased [Ca^2+^]_I_ in EEC induced by proteases [12]. Ruan and colleagues stipulated the role of ENaC and VDCC in the protease-activated Ca^2+^ signaling. Activation of ENaC via trypsin would lead to Na^+^ influx, membrane depolarization, activation of VDCC and ultimately the desired Ca^2+^ influx. Although the expression of ENaC in EEC of mouse and human could be confirmed via RNAscope and RT-qPCR experiments, only a single subunit of VDCC (*Ca*_*v*_* 1.2*) could be detected. Moreover, inhibition of ENaC by amiloride and of VDCCs by nifedipine did not abolish the trypsin-induced [Ca^2+^]_I_ response. We did observe that the number of responding cells was decreased upon amiloride application. However, removal of external Na^+^ did not alter trypsin-induced [Ca^2+^]_I_ oscillations in mEEC, excluding the potential contribution of ENaC in the [Ca^2+^]_I_ influx. Possibly, the effect of amiloride on trypsin responses may reflect direct modulation of GPCRs by amiloride, as has been described in earlier studies, especially for the class A GPCRs to which PAR members belong [32, 33]. In addition, the lack of voltage-induced currents by whole-cell patch clamp experiments argued against the functional presence of VDCC in mEEC. Furthermore, RNA expression data confirmed the expression of ENaC in EEC of mouse and human, but could not implicate a role for ENaC in [Ca^2+^]_I_ influx induced by trypsin. Another recent study proposed PAR1 and PAR2 as important receptors to regulate the trypsin-induced endometrial [Ca^2+^]_I_ signaling in Ishikawa cells [13]. Very low gene counts were detected for *F2r* (PAR1) in both mouse and human EEC, arguing against the potential contribution of PAR1 in the trypsin-induced [Ca^2+^]_I_ oscillations. In addition, it was proposed that Na^+^ entry via trypsin-activated ENaC would depolarize the cellular membrane and increase the intracellular Na^+^ concentration to reverse the sodium calcium exchanger (NCX) thereby providing means for Ca^2+^ entry [13]. However, *Scl8a1* expression in EEC was low, and no change in [Ca^2+^]_I_ was detected upon switching to Na^+^-free solution, suggesting a minor contribution of the NCX in the [Ca^2+^]_I_ influx.

Strong evidence is provided for the involvement of PAR2 in trypsin-induced [Ca^2+^]_I_ oscillations in EEC. First, high gene counts were detected for *F2rl1* (PAR2) in EEC of mouse and human. Moreover, in situ hybridization and RT-qPCR experiments further confirmed this molecular presence of *F2rl2* (PAR2) in different cellular models of mouse and human. Next, functional expression of PAR2 was shown by the use of a selective PAR2 agonist, 2-fu, which could trigger a comparable [Ca^2+^]_I_ response in EEC of mouse and human as trypsin. Additionally, application of the PAR2 antagonist, I-191 reversibly inhibited [Ca^2+^]_I_ oscillations induced by either trypsin or 2-fu_._ Finally, these findings were confirmed in excised uterine tissue fragments via ex vivo Ca^2+^ imaging experiments, where trypsin or 2-fu were able to induce [Ca^2+^]_I_ oscillations. Spontaneous [Ca^2+^]_I_ oscillations could also be observed in the control condition of bath solution without perfusion, which could be explained by the induction of mechanical stimulation of the tissue during experimental handlings prior to the fluorescent measurements (e.g. cutting and tightening of the uterus). Furthermore, our study unraveled part of the underlying molecular mechanism of trypsin-induced [Ca^2+^]_I_ influx in EEC. The [Ca^2+^]_I_ oscillations are mainly caused via Ca^2+^ release from and re-uptake into intracellular stores, often effectuated via PLC activation, generation of IP_3_ and inducement of store operated calcium entry (SOCE). However, at the moment it is unclear what the underlying oscillator is and whether these [Ca^2+^]_I_ oscillations also induce oscillations of the membrane potential. Ca^2+^ microfluorimetric experiments showed a strong reduction of the number of oscillating EEC when trypsin was coapplied in the presence of specific inhibitors of PLC, IP_3_R, SERCA and CRAC. A previous study in Ishikawa cells proposed a similar mechanism to sustain the trypsin-induced Ca^2+^ entry via STIM1/Orai1 store operated Ca^2+^ channels [13]. However, a detailed investigation of the signaling pathway in primary culture of mEEC was never shown before. The positive effect of shear stress on the [Ca^2+^]_I_ oscillations could potentially be explained by the functional expression of mechanosensitive ion channels in primary endometrial epithelial cells, like PIEZO1 [21]. The co-application of shear stress and trypsin, potentiated the percentage of oscillating cells, which could be explained by a combined effect of mechanosensitive channels and PAR2. Recently, a study showed the interaction between GPCR and PIEZO1 channel [34]. It was suggested that upon GPCR activation, the dissociation of Gβγ associated protein could potentially also modulate PIEZO1 activity. Additional research is required to disentangle the potential link between PAR2 and the mechanosensitive PIEZO1.

Next, the physiological impact of PAR2-induced [Ca^2+^]_I_ oscillations was assessed during pre-decidualization in human EEC. Interestingly, EEC derived from human showed a significant upregulation of the WOI markers after incubation with the PAR2 agonist, 2-fu. These results indicate that PAR2-induced [Ca^2+^]_I_ influx potentially play an important role in the regulation of the decidualization of stromal cells, which is key for successful implantation. At the moment additional research is required to further explore the in vivo role of PAR2 activation in the implantation process. Earlier studies did not show major fertility problems in full body PAR2 knock-out mice at first sight [35]. However, smaller litter sizes were observed together with increased embryonic lethality [36], which was even more pronounced in combination with PAR1 deletion [37]. Altogether, these results suggest that deletion of PAR2 could be linked to a phenotype in reproduction. Of course, it cannot be excluded that compensation by other receptors occurs in the full body PAR2-/- mice as was suggested by [35], obscuring possible reproductive phenotypes by single PAR2 deletion. Further investigation, especially into early implantation events, is warranted to generate a conclusive decision concerning PAR2 fertility in vivo. Furthermore, our observations are in line with previously published research that showed that medium obtained from developmentally competent human embryos is able to induce [Ca^2+^]_I_ responses in Ishikawa cells [8]. These [Ca^2+^]_I_ responses were short-lived and organized, whereas [Ca^2+^]_I_ responses induced with conditioned medium obtained from developmentally incompetent embryos were lasting and chaotic. It was speculated that [Ca^2+^]_I_ patterns generated by the embryo-derived proteases would convey embryo quality towards the maternal endometrium, allowing for implantation of competent blastocysts only. Our data illustrate the presence of oscillatory Ca^2+^ signals in EEC induced by embryonic signals released by the outgrowing trophoblast cells. Potentially, PAR2-induced [Ca^2+^]_I_ oscillations are important for the selection of competent blastocysts, in which a lack of [Ca^2+^]_I_ oscillations or an overload of [Ca^2+^]_I_ would influence further steps of the implantation process in human, like stromal decidualization and embryo selection.

## Conclusion

Our research focused on the effects of blastocyst-derived serine proteases on maternal EEC, providing novel insights in the molecular players and Ca^2+^ signaling pathways during the embryo-uterine crosstalk. These results show the ability of primary EEC to generate [Ca^2+^]_I_ oscillations upon contact with trypsin, a process dependent on Ca^2+^ release via the PLC/IP_3_R/SOCE pathways. Evidence is provided for PAR2 as initiator of [Ca^2+^]_I_ responses and potential regulator of pre-decidualization events in human. These findings could lead to novel clinical applications to the further optimization of the IVF-protocol to improve IVF success rates. However, additional research is required to further unravel the exact role of PAR2 in the early implantation events in human.

## Supplementary Information

**Additional file 1: Supplementary Fig. 1.** Progesterone-induced uterine gland knock-out model. **Supplementary Fig. 2.** Trypsin induced calcium oscillations in endometrial epithelial cells. **Supplementary Fig. 3.** Expression of Enac in mEEC. **Supplementary Fig. 4.** Aprotinin has no effect on the 2-fu induced [Ca^2+^]_I_ oscillations. **Supplementary Fig. 5.** Stromal cells do not show [Ca^2+^]_I_ oscillations after stimulation by trypsin. **Supplementary Fig. 6.** Ex vivo Ca^2+^ imaging in excised uterine tissue. **Supplementary table 1.** overview of used RNAscope probes and RT-PCR assays.

**Additional file 2.**

**Additional file 3.**

**Additional file 4.**

**Additional file 5.**

## Data Availability

The datasets used and/or analyzed during the current study are available from the corresponding author on reasonable request.

## Abbreviations

2-fu: 2-Furoyl-LIGRLO-NH2
Am: Amiloride
Apr: Aprotinin
BSA: Bovine serum albumin
cAMP: 3’,5’-Cyclic adenosine monophosphate (Cyclic AMP)
COX-2: Cyclooxygenase-2
CRAC: Calcium Release Activated Channel
CREB: CAMP response element binding protein
CXCL14: C-X-C motif chemokine 14
DAPI: 4′,6-Diamidino-2-phenylindole
DMEM: Dulbecco modified Eagle’s medium
DMSO: Dimethyl sulfoxide
dpf: Day post fertilization
E2: 17-β-Estradiol
Ecad: Epithelial cadherin
EEC: Endometrial Epithelial Cell
ENaC: Epithelial Sodium channel
ER: Endoplasmic Reticulum
ESC: Endometrial Stromal Cell
FBS: Fetal bovine serum
FOXA2: Forkhead box A2
GAPDH: Glyceraldehyde-3-phosphate dehydrogenase
GPCR: G-Protein Coupled Receptor
GPX3: Glutathione peroxidase homolog 3
hEEC: Human endometrial epithelial
hEMO: Human endometrial epithelial organoids
hESC: Human stromal epithelial
HPRT1: Hypoxanthine phosphoribosyl transferase 1
ICM: Inner cell mass
Iono: Ionomycin
IP_3_: Inositol 1,4,5-trisphosphate
IP_3_R: IP_3_ receptor
ISH: In situ hybridization
IVF: In Vitro Fertilization
LIF: Leukemia Inhibitory Factor
mEEC: Mouse endometrial epithelial
mESC: Mouse stromal epithelial
mRNA: Messenger RNA
NCX: Sodium calcium exchanger
ORAI1: Calcium Release-Activated Calcium Modulator 1
P4: Progesterone
PAEP: Progestagen-associated endometrial protein
PAR: Protease-Activated Receptor
PBS: Phosphate Buffered Saline
PFA: Paraformaldehyde
PGE2: Prostaglandin E2
Pgk1: Phosphoglycerate Kinase 1
PLC: Phospholipase C
PR: Progesterone receptor
PUGKO: Progesterone-induced uterine gland knockout
RT: Room temperature
RT-qPCR: Real-Time quantitative Polymerase Chain Reaction
SD: Standard deviation
SEM: Standard error of the mean
SERCA: Sarco/endoplasmic reticulum Ca^2+^ ATPase
SOCE: Store-Operated Calcium Entry
SPP1: Secreted Phosphoprotein 1
SS: Shear stress
STIM: Stromal interaction molecule
Tbp: TATA-Box Binding Protein
TBS: TRIS-buffered saline
TE: Trophectoderm
Thapsi: Thapsigargin
Tryp: Trypsin
Trpv6: Transient Receptor Potential Cation Channel Subfamily V Member 6
UMAP: Uniform manifold approximation and projection
VDCC: Voltage-Dependent Calcium Channel
WOI: Window of implantation
