# Supplementary material for: Protease secretions by the invading blastocyst induce calcium oscillations in endometrial epithelial cells via the protease-activated receptor 2
Source: Reprod Biol Endocrinol. 2023 Apr 15;21:37. doi: 10.1186/s12958-023-01085-7 (PMC10105462; doi:10.1186/s12958-023-01085-7)
Supplement: Supplementary file 1 — Additional file 1: Supplementary Fig. 1. Progesterone-induced uterine gland knock-out model. Supplementary Fig. 2. Trypsin induced calcium oscillations in endometrial epithelial cells. Supplementary Fig. 3. Expression of Enac in mEEC. Supplementary Fig. 4. Aprotinin has no effect on the 2-fu induced [Ca2+]I oscillations. Supplementary Fig. 5. Stromal cells do not show [Ca2+]I oscillations after stimulation by trypsin. Supplementary Fig. 6. Ex vivo Ca2+ imaging in excised uterine tissue. Supplementary table 1. overview of used RNAscope probes and RT-PCR assays. [file 12958_2023_1085_MOESM1_ESM.docx]

**Protease secretions by the invading blastocyst induce calcium oscillations in endometrial epithelial cells via the protease activated receptor-2**

Aurélie Hennes^1,2,#^ Johanna Devroe^1,2,3, #^, Katrien De Clercq^1,2^, Martina Ciprietti^1,2^, Katharina Held^1,2^, Katrien Luyten^1^, Nele Van Ranst^2^, Nina Maenhoudt^4^, Karen Peeraer^1,3^, Hugo Vankelecom^4^, Thomas Voets^2^ and Joris Vriens^1,*^

**Supplementary Information**

# SUPPLEMENTARY FIGURES

# Supplementary figure 1


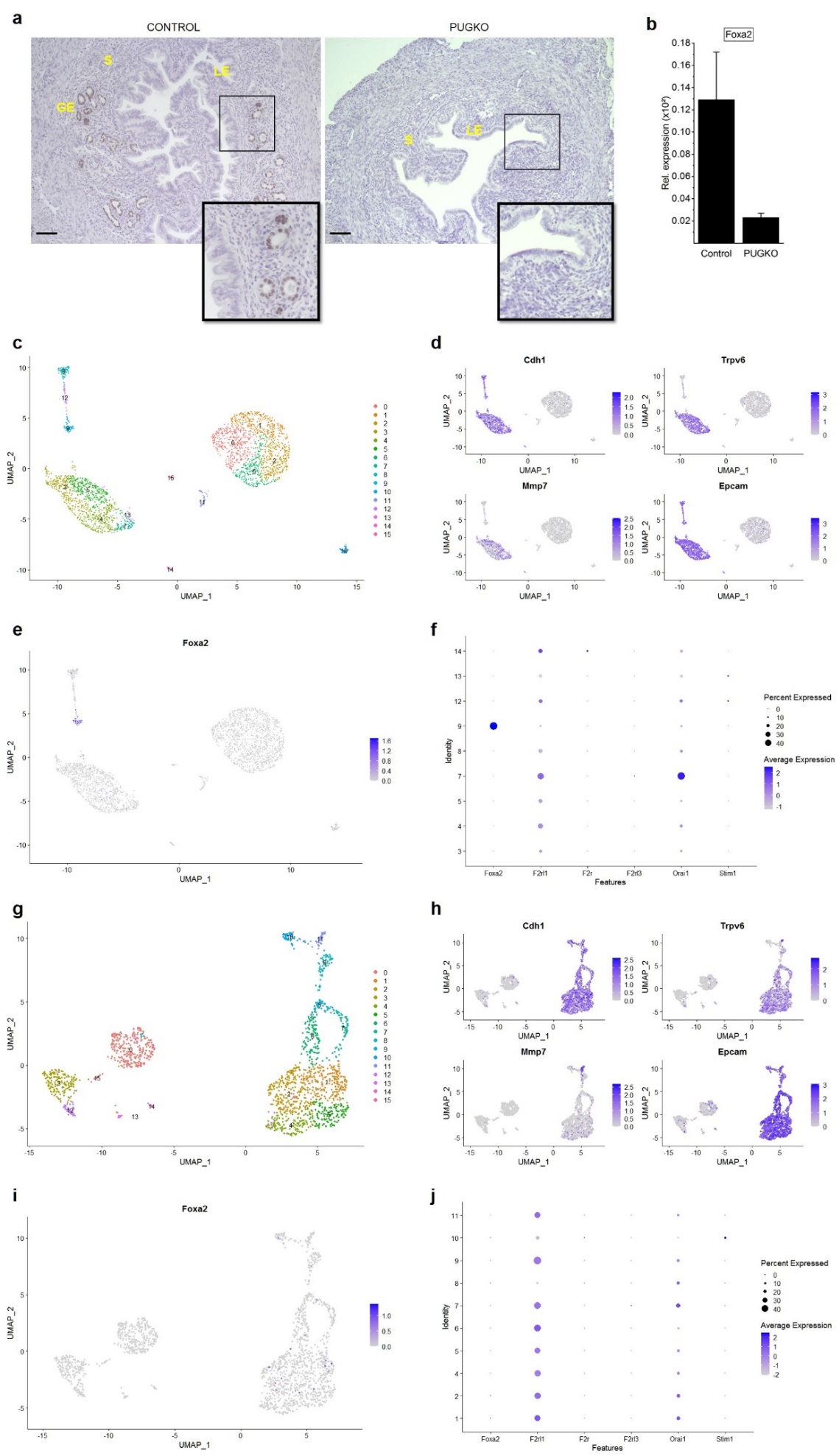


**Supplementary Figure 1: Progesterone-induced uterine gland knock-out model**

**(A)** Representative images of FOXA2 staining in uterine sections derived from control or PUGKO animals. The boxes in the lower right corner are magnifications of the indicated areas. Scale bar: 50 µm. LE: luminal epithelium, GE: glandular epithelium, S: stroma. **(B)** RT-qPCR expression levels for *Foxa2* in isolated control or PUGKO epithelial cells. Expression is relatively quantified compared to geometric mean of the housekeeping genes *Tbp* and *Pgk1*. Data is shown as mean ± SEM. **(C, G)** Uniform manifold approximation and projection (UMAP) of the distinct epithelial populations within isolated mEEC from control (C) or PUGKO (G) animals. **(D, H)** Expression of different epithelial markers, *Cdh1* (*E-cadherin*), *Trpv6*, *Mmp7* and *Epcam*, in UMAP space in the cell population described under (C) and (G) respectively. **(E, I)** Evaluation of *Foxa2* expression in UMAP space for the cell population described under (C) and (G) respectively. **(F, J)** Dot plot representing the average expression and expression percentage of several genes in the isolated mEEC from control (F) and PUGKO (J) animals.

# Supplementary figure 2


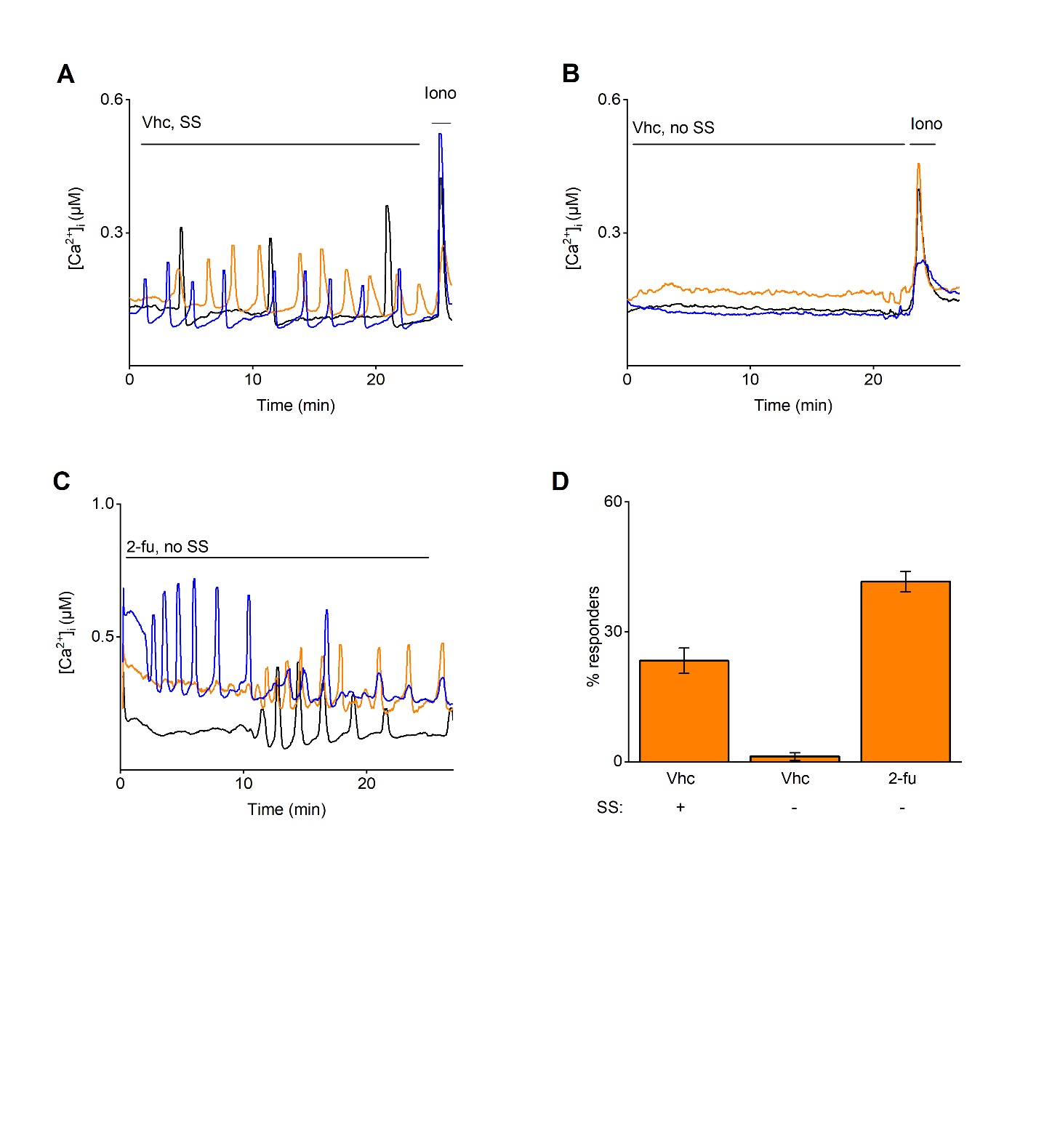


**Supplementary Figure 2. Trypsin induced calcium oscillations in endometrial epithelial cells**

Ca^2+^ microfluorimetry. **(A-B)** depict representative traces of mouse EEC subjected to the bath solution without additional compounds (vehicle), in the presence (A) or absence (B) of shear stress. In **(C)** representative traces are shown of 2-furoyl-LIGRLO-NH_2_ stimulation without shear stress. **(D)** Percentage of oscillating cells (≥ 2 intracellular Ca^2+^ peaks) to stimulation in Ca^2+^ microfluorimetric experiments with vehicle, 2-furoyl-LIGRLO-NH_2_ (5 µM) in the presence (+) or absence (-) of shear stress. Responders are shown as mean ± SEM. Three representative traces are shown in each graph. Ionomycin (2 µM) was added as a positive control at the end of each experiment. n = at least independent 3 experiments with a total minimum of 200 cells per condition.

Tryp = trypsin, Iono = ionomycin, 2-fu = 2-furoyl-LIGRLO-NH_2_, SS = shear stress, vhc = vehicle.

# Supplementary figure 3


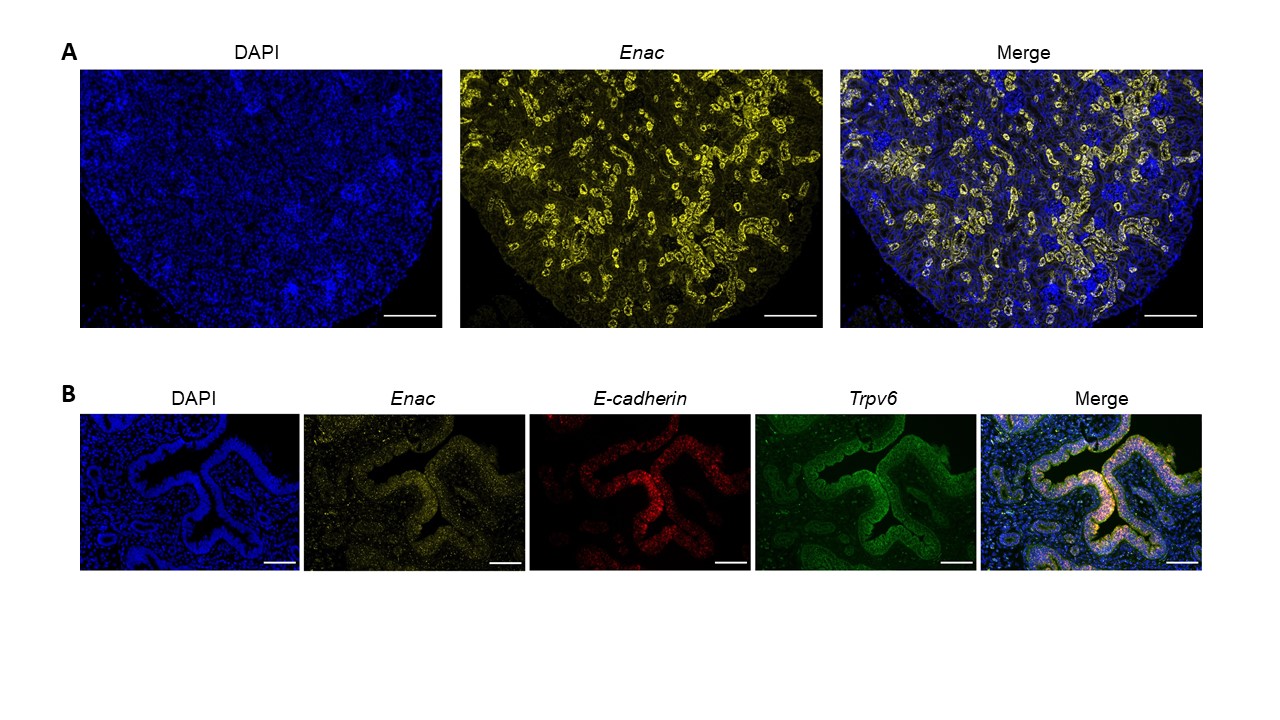


**Supplementary Figure 3. Expression of Enac in mEEC**

**(A)** In situ hybridization RNAscope images of kidney tissue sections. Positive signals were detected for the *EnaC*. Scale bar: 100 µm. **(B)** In situ hybridization RNAscope images of uterine tissue sections. Modest signals were detected for the epithelial markers *E-cadherin* and *Trpv6*. Limited signals could be observed for *Enac*. Scale bar: 50 µm.

# Supplementary figure 4


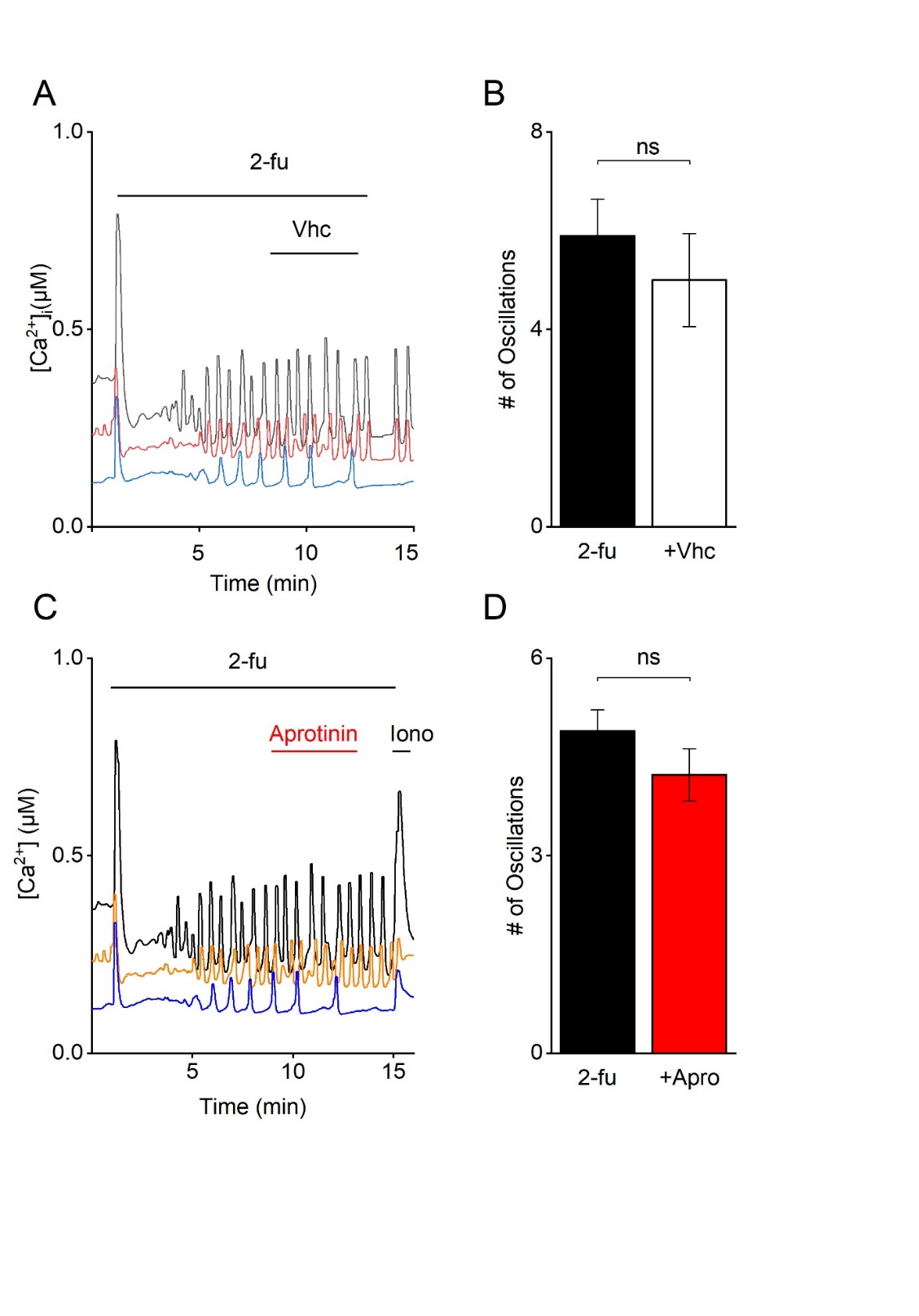


**Supplementary Figure 4. Aprotinin has no effect on the 2-fu induced [Ca^2+^]_I_ oscillations**

Ca^2+^ microfluorimetry. **(A)** representative traces of mEEC stimulated with 2-fu (5 µM) in the absence (vhc) of presence **(C)** of aprotinin (20 µg/ml). **(B)** en **(D)** represent the number of oscillating cells (≥ 2 intracellular Ca^2+^ peaks) for the experiments performed under (A) en (C) respectively. Responders are shown as mean ± SEM. Ionomycin (2 µM) was added as a positive control at the end of each experiment. n = at least independent 3 experiments with a total minimum of 200 cells per condition.

Apro = aprotinin, Iono = ionomycin, 2-fu = 2-furoyl-LIGRLO-NH_2_, Vehicle (vhc) = bath solution without additional compounds, ns = not significant.

# Supplementary figure 5


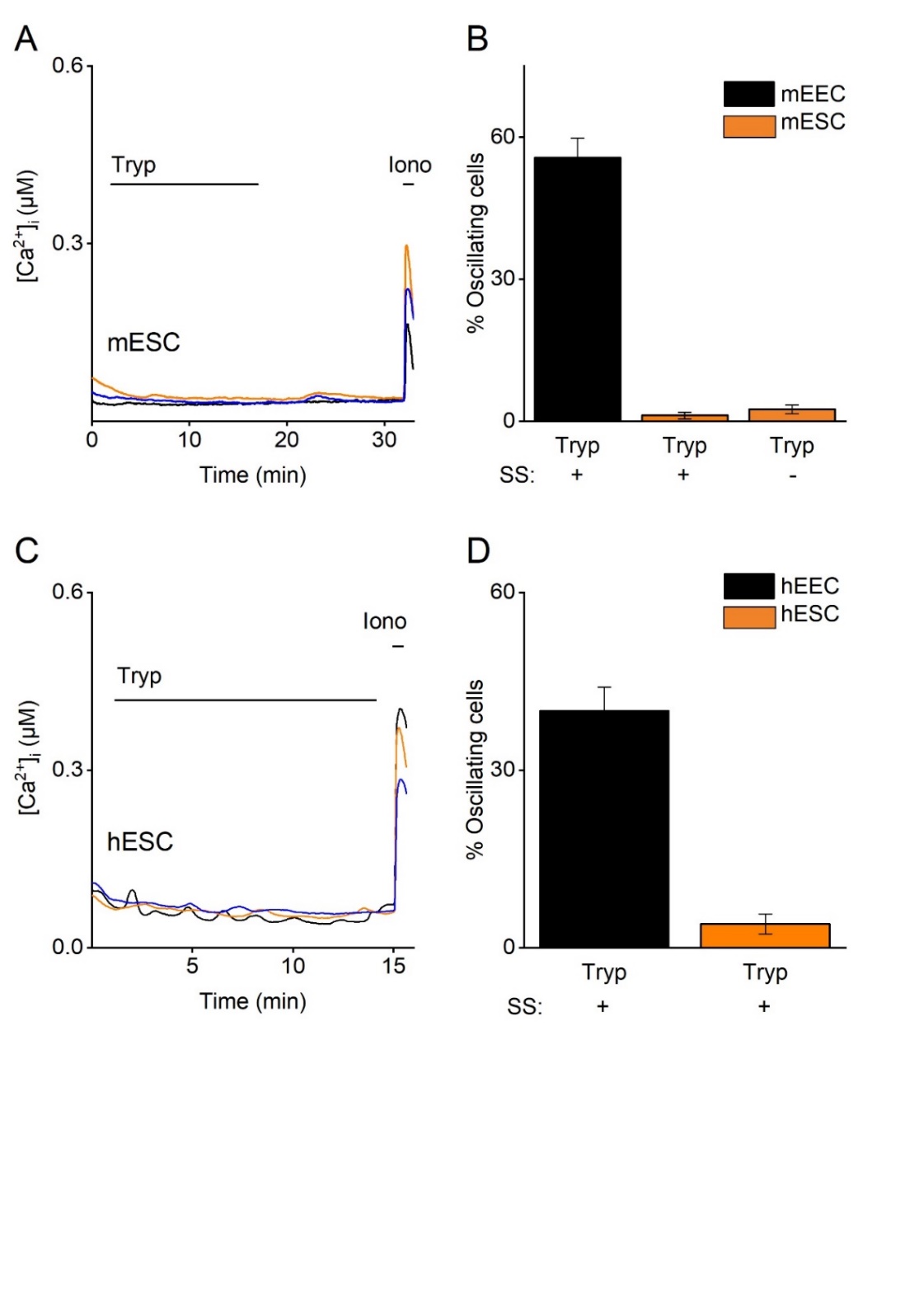


**Supplementary Figure 5: Stromal cells do not show [Ca^2+^]_I_ oscillations after stimulation by trypsin.**

Ca^2+^ microfluorimetry. (A) Three representative traces of mESC subjected to trypsin. (B) Percentage of oscillating cells (≥ 2 intracellular Ca2+ peaks) of mEEC and mESC to stimulation with trypsin (2 µg/ml) in the presence (+) or absence (-) of shear stress. Responders are shown as mean ± SEM.(C) Three representative traces of hESC subjected to trypsin. (D) Percentage of oscillating cells (≥ 2 intracellular Ca2+ peaks) of hEEC and hESC to stimulation with trypsin (2 µg/ml). Responders are shown as mean ± SEM. Ionomycin (2 µM) was added as a positive control at the end of each experiment. n = at least 3 independent experiments with a total minimum of 200 cells per condition.

Tryp = trypsin, Iono = Ionomycin

# Supplementary figure 6


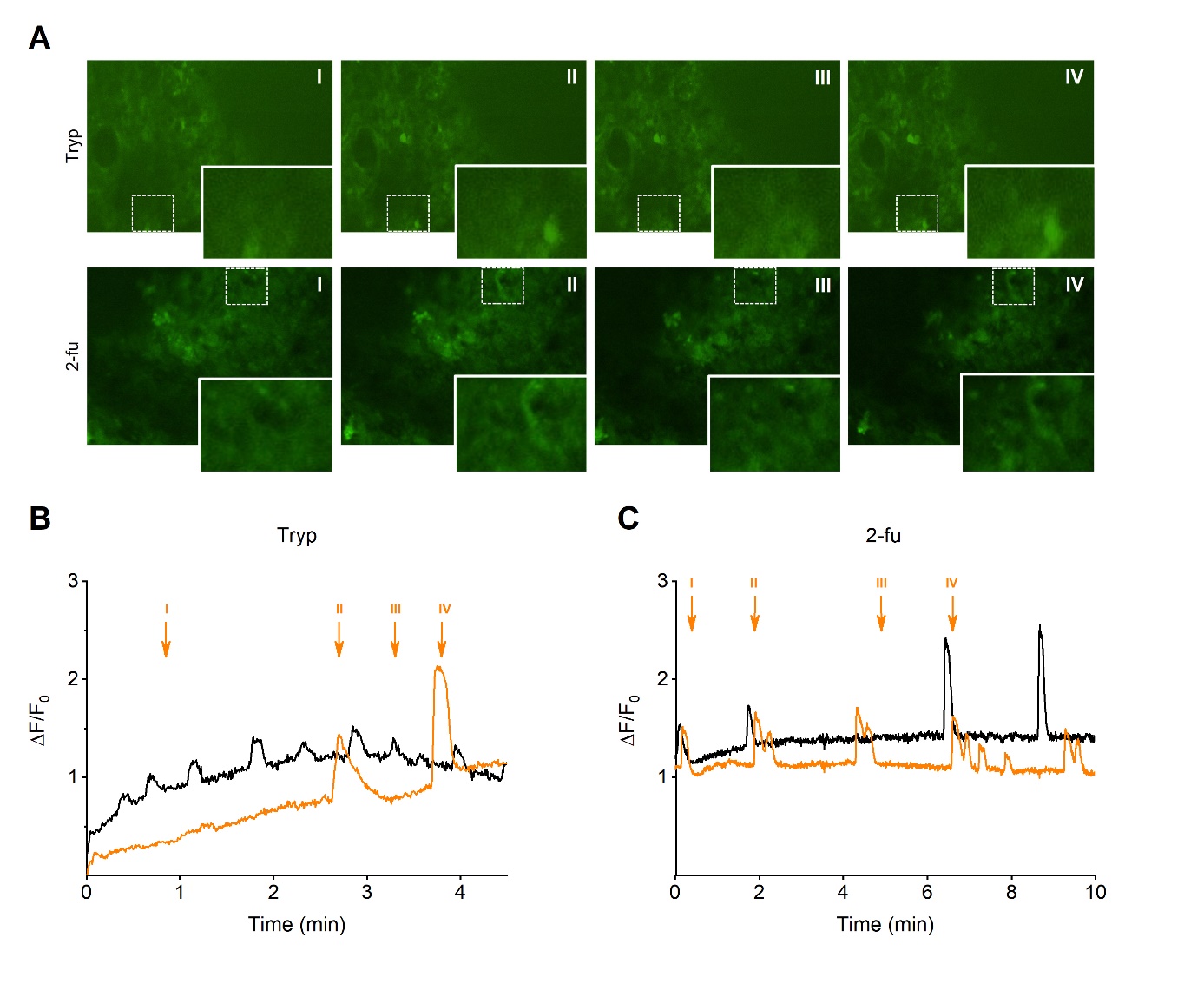


**Supplementary Figure 6. *Ex vivo* Ca^2+^ imaging in excised uterine tissue**

**(A)** Still frame pictures extracted from *ex-vivo* Ca^2+^ imaging on excised uterine tissues. Stimulation with trypsin (2 µg/ml) (top panels) or 2-furoyl-LIGRLO-NH_2_ (5 µM) (bottom panels) is shown. Dotted boxes indicate a specific cell of which the intracellular Ca^2+^ concentration was shown in (B) or (C). The area represented by the dotted box is enlarged in the lower right panels. **(B, C)** depict two representative intracellular Ca^2+^ traces when stimulated with trypsin or 2-furoyl-LIGRLO-NH_2_. The trace in orange corresponds with the frames in (A). Arrows indicate the time points when the frames were extracted. Tryp = trypsin, 2-fu = 2-furoyl-LIGRLO-NH_2_

# SUPPLEMENTARY MOVIES

***Supplementary Movie 1***

<https://youtu.be/lL7mc-1Fr0k>

***Supplementary Movie 2***

<https://youtu.be/pC9-dABf75Y>

***Supplementary Movie 3***

<https://youtu.be/DE-dF9u9pG8>

***Supplementary Movie 4***

<https://youtu.be/fRaO3LN4fNw>

# SUPPLEMENTARY TABLE

| **RNAscope probes** | | |
| --- | --- | --- |
| **Product** | **Company** | **Product number** |
| Human CDH1 | ACDBio | 311091 |
| Human F2RL1 | ACDBio | 319861 |
| Mouse F2rl1 | ACDBio | 417541 |
| Mouse Cdh1 | ACDBio | 408651 |
| Mouse TrpV6 | ACDBio | 539131 |
| **RT-qPCR gene expression assays** | | |
| **Product** | **Company** | **Assay ID** |
| Mouse F2rl1 | Life Technologies | Mm00433160_m1 |
| Mouse Orai1 | Life Technologies | Mm00774349_m1 |
| Mouse Stim1 | Life Technologies | Mm01158413_m1 |
| Mouse Tbp | Life Technologies | Mm01277042_m1 |
| Mouse Pgk1 | Life Technologies | Mm00435617_m1 |
| Mouse Foxa2 | Life Technologies | Mm01976556_s1 |
| Mouse Scnn1a | Life Technologies | Mm00803386_m1 |
| Mouse Cacna1c | Life Technologies | Mm01188812_m1 |
| Mouse Cacna1d | Life Technologies | Mm01209927_g1 |
| Mouse Cacna1f | Life Technologies | Mm01352612_m1 |
| Human F2RL1 | Life Technologies | Hs00608346_m1 |
| Human ORAI-1 | Life Technologies | Hs03046013_m1 |
| Human STIM1 | Life Technologies | [Hs00963373_m1](https://www.thermofisher.com/taqman-gene-expression/product/Hs00963373_m1?CID=&ICID=&subtype=) |
| Human PGK1 | Life Technologies | Hs00943178_g1 |
| Human HPRT1 | Life Technologies | Hs02800695_m1 |

**Supplementary table 1: overview of used RNAscope probes and RT-PCR assays.**
